# Rare Earth Ce/CeO_2_ Electrocatalysts: Role of High Electronic Spin State of Ce and Ce^3+^/Ce^4+^ Redox Couple on Oxygen Reduction Reaction

**DOI:** 10.3390/nano15080600

**Published:** 2025-04-14

**Authors:** Shaik Gouse Peera, Seung Won Kim

**Affiliations:** Natural Science Research Institute, College of Natural Sciences, Keimyung University, 1095 Dalgubeol-daero, Daegu 42601, Republic of Korea; swkim@kmu.ac.kr

**Keywords:** oxygen reduction reaction, rare-earth metal catalysts, oxygen vacancies, CeO_2_, fuel cells, atomically dispersed Ce, radical scavenging, high-spin Ce electrocatalysis

## Abstract

With unique *4f* electronic shells, rare earth metal-based catalysts have been attracting tremendous attention in electrocatalysis, including oxygen reduction reaction (ORR). In particular, atomically dispersed Ce/CeO_2_-based catalysts have been explored extensively due to several unique features. This review article provides a comprehensive understanding of (i) the significance of the effect of Ce high-spin state on ORR activity enhancement on the Pt and non-pt electrocatalysts, (ii) the spatially confining and stabilizing effect of ceria on the generation of atomically dispersed transition metal-based catalysts, (iii) experimental and theoretical evidence of the effect of Ce^3+^ ↔ Ce^4+^ redox pain on radical scavenging, (iv) the effect of the Ce 4f electrons on the d-band center and electron transfer between Ce to the N-doped carbon and transition metal catalysts for enhanced ORR activity, and (v) the effect of Pt/CeO_2_/carbon heterojunctions on the stability of the Pt/CeO_2_/carbon electrocatalyst for ORR. Among several strategies of synthesizing Ce/CeO_2_ electrocatalysts, the metal–organic framework (MOF)-derived catalysts are being perused extensively due to the tendency of Ce to readily coordinate with O- and N-containing ligands, which upon undergoing pyrolysis, results in the formation of high surface area, porous carbon networks with atomically dispersed metallic/clusters/nanoparticles of Ce active sites. This review paper provides an overview of recent advancements regarding Ce/CeO_2_-based catalysts derived from the MOF precursor for ORR in fuel cells and metal–air battery applications and we conclude with insights into key issues and future development directions.

## 1. Introduction

Increasing global warming due to the extensive use of carbon-based fossil fuels escalating environmental concerns has led to a demand for alternative technologies to meet the growing demand of energy [1,2]. The electrochemical oxygen reduction reaction (ORR) is a central, cathodic electrochemical reaction which plays a critical role in various energy conversion devices like fuel cells and metal–air batteries like Zn–air and Al–air batteries [3,4,5]. In these devices, the ORR occurs on the cathodes, where the electrocatalysts reduce the atmospheric oxygen into H_2_O/OH^−^. Due to the process being inherently sluggish and requiring multiple steps, kinetically controlled ORR often requires efficient electrocatalysts to enhance effective electron transfer and the reduction of O_2_ [6,7]. In general, the best state-of-the-art electrocatalysts for ORR are Pt nanoparticles supported by high surface-area carbon support (Pt/C) [8]. Despite the best ORR activity of Pt/C catalysts, successful commercial application of the energy conversion devices is severely hindered due to the high cost and scarcity of Pt, together with the poor stability of the Pt/C catalysts [9]. It is well known that a commercial Pt/C catalyst undergoes a variety of degradation pathways, such as electrochemical carbon corrosion, Pt nanoparticle dissolution and agglomeration, catalyst poisoning in the presence of gasses such as CO and SO_x_, etc. Pt nanoparticles detach from the carbon support due to the loss of supported solid carbon due to carbon corrosion [10]. Consequently, research is being conducted to develop effective and stable electrocatalysts, which incorporate stable supports to enhance catalyst stability, reducing the Pt loading, as well as cost-effective catalysts utilizing transition and inner-transition metals, including lanthanides, to diminish dependence on costly platinum-based materials [9,11]. To substantially decrease the quantity of Pt loading, many solutions have been implemented, such as alloying Pt with other metals. The noble Pt metal have been alloyed with various transition metals (such as Fe, Co, Ni, Mn, Cu, etc.) and inner-transition metals of lanthanides (such as, Ce, La, Y, Gd, Dy, etc.) and it was found that Pt-alloy catalysts exhibit excellent ORR activity over traditional Pt/C catalysts [12,13,14]. However, even the Pt-alloys catalysts also suffer from issues like dissolution, Ostwald ripening, and nanoparticle sintering. In addition, the Pt-alloy catalysts show poor stability, suggesting the need for active and stable electrocatalysts for ORR both in acidic and alkaline electrolytes [15].

Though basic and advanced research on Pt, Pt-alloy, and non-precious metal catalysts with active sites composed of M_x_-N_y_-C (M=TM, such as Fe, Co, Mn, Cu, Ni, etc.) is so popular, with tens to thousands of papers being published each month [16,17], it is very recently that research exploring the possibility of using inner-transition metals of lanthanides from the *f*-block series has been attracting tremendous attention [18]. Rare earth elements are composed of 17 elements: lanthanum (La), cerium (Ce), praseodymium (Pr), neodymium (Nd), promethium (Pm), samarium (Sm), europium (Eu), gadolinium (Gd), terbium (Tb), dysprosium (Dy), holmium (Ho), erbium (Er), thulium (Tm), ytterbium (Yb), lutetium (Lu), scandium (Sc), and yttrium (Y). While rare earth elements are not great electrocatalysts on their own, even in trace amounts they can change the characteristics of active sites composed of other noble and transition metals [19]. Rare earth metals find several applications in day-to-day life, for example, several rare earth metal oxides have been used as polishing powders in the making of glass products with various rare earth metal oxides. The quality of gasoline is also improved by using catalysts that contain rare earth elements [20]. Cerium oxide is used in the three-way catalytic converter of waste gas treatment systems to change toxic waste gas into a harmless gas that people can breathe in. Due to their use and applications in various industrial products, today, rare earth elements are termed “Industrial Gold” or “Industrial Vitamins” [21]. Rare-earth (RE) elements exhibit various oxidation states such as RE^2+^, RE^3+^, and RE^4+^, of which RE^3+^ is the most common. The presence of these various oxidation states suggests that RE with empty, half-filled, and fully filled electron energy levels tends to be more stable, as suggested by Hende’s rule [21]. Rare earth elements are specially characterized by the presence of unique *f*-orbitals. In general, 4*f* electrons are inert, meaning they do not participate in chemical bonding with other elements and are part of the ionic core of the element. However, the positioning and incomplete filling of 4f electrons gives rare earth elements unique physical and chemical properties that are suitable for electrocatalytic applications [22,23]. In addition, the rare earth metallic elements exhibit strong coordination ability with reactants’ molecular orbitals and as rare earth metal oxides have the features of oxygen affinity and oxygen vacancies, in various catalytic reactions, rare earth oxides act as co-catalysts by transferring the stored oxygen species and hence promote higher catalytic activity of the main catalysts [24,25].

Among the various RE elements, cerium (Ce)-based catalysts, either in an atomically dispersed state or in the form of Ce-oxide (CeO_2_), has attracted considerable attention for various electrochemical reactions such as CO oxidation, NO reduction, water–gas-shift reaction, and for ORR [26]. The rich oxygen vacancies and affinity and facile interchange of the oxidation state of Ce^3+^ and Ce^4+^ facilitates the ORR. In view of the fuel cell reaction, the ability to perform the interexchange of Ce^3+^/Ce^4+^ in the oxidation state is so important; for this, it is essential that Ce inactivates the hydrogen peroxide, formed from the 2-electron O_2_ reduction reaction. CeO_2_ has high stability under acidic conditions and CeO_2_ also improves the corrosion-resistant ability of carbon in acidic electrolytes [27]. CeO_2_ also exhibits strong interaction with noble metals such as Pt and non-precious metals, thus enhancing the metal/metal oxide interactions in promoting the ORR by facilitating optimal adsorption of O_2_ and ORR intermediates [28]. Furthermore, the 4f orbit of the Ce atom allows for both unoccupied and one-electron occupied states, and the 4f shell of the Ce atom is suitable for electron sharing and bonding [29]. Furthermore, ceria plays a key function in promoting the dispersion of noble metals; ceria can significantly enhance O_2_ adsorption and ORR kinetics. Ceria-modified carbon compounds have been shown to serve as very efficient catalysts for a direct 4-electron reduction in ORR [30,31]. These distinct features of ceria motivated us to search for the possibility of Ce/CeO_2_ materials being employed as ORR catalysts. This review paper provides an overview of the research that had been carried out recently on the applications of Ce and CeO_2_ for ORR for fuel cells and metal–air batteries. The first part of this review paper describes the characteristics and properties of Ce/CeO_2_ and the second part of the review paper describes the ORR kinetics of the electrocatalysts of Ce/CeO_2_ alone or in combination with Pt and non-Pt metallic active sites, derived from metal–organic framework (MOF) precursors. MOFs are a class of porous materials comprising metal ions/organic linkers; after undergoing pyrolysis, they result in the formation of a porous carbon network with atomically dispersed metallic/clusters/nanoparticles of active sites [32].

MOF-derived catalysts have recently gained tremendous interests regarding their application in various fields, including gas storage and separation, luminescence, magnetism, biology and medicine, biomass conversion, photovoltaic cells, water purification, and catalysis; this includes ORR [33]. Due to the tendency of RE metals to readily coordinate with O- and N-containing ligands, particularly -COOH ligands, together with their high coordination numbers and varied coordination modes, the synthesis of novel structural metal–organic frameworks (MOFs) is feasible. Nevertheless, owing to steric hindrance, rare earth elements are unable to fully coordinate with ligands, resulting in one or more coordination sites being available to interact with solvent molecules, thereby forming end groups. Upon heat treatment of the synthesized rare earth MOFs, solvent molecules are expelled from the rare earth MOF, revealing coordinatively unsaturated sites within the structure that can serve as active Lewis’s acid/base centers, that can interact with reactant species like O_2_ to promote the ORR [34]. Rare earth ions possess unsaturated 4f orbitals and readily coordinate with the lone electron pairs of organic groups. The ligands most commonly used for the synthesis of RE MOFs include terephthalic acid; 1,4-benzene dicarboxylic acid (BDC); trimesic acid (TMA), also known as 1,3,5-benzenetricarboxylic acid (H_3_BTC); pyridine-2,4-dicarboxylic acid (2,4-pydc); 2-aminoterephthalic acid (BDC-NH_2_); zeolite imidazole framework (ZIF); Zn-triazolates; Prussian blue; and various other organic ligands [35].

**Figure 1:**
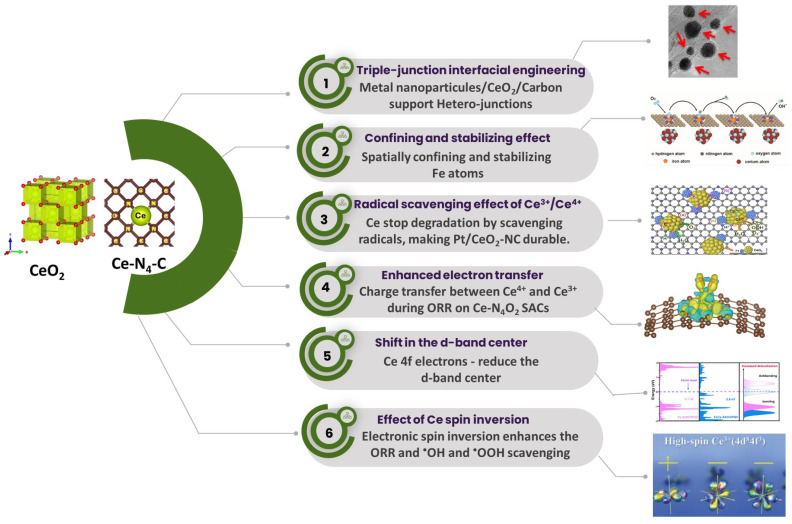
The unique properties of the Ce/CeO_2_-based electrocatalysts for ORR.

To briefly conclude, this review article provides a comprehensive understanding of (i) the significance of Ce high-spin state on the ORR activity enhancement on the Pt and non-pt electrocatalysts, (ii) the spatially confining and stabilizing effect of ceria on the generation of the atomically dispersed transition metal-based catalysts, (iii) experimental and theoretical evidence of the effect of Ce^3+^ ↔ Ce^4+^ redox pain on radical scavenging, (iv) the effect of the Ce 4f electrons on the d-band center and electron transfer between Ce to the N-doped carbon and transition metal catalysts for enhanced ORR activity, and (v) the effect of Pt/CeO_2_/carbon heterojunctions on the stability of the Pt/CeO_2_/carbon electrocatalyst for ORR (Figure 1).

## 2. Properties of Cerium Dioxide/Atomically Dispersed Cerium for ORR

The element cerium (Ce), which has the atomic number 58, is by far the most abundant element from the group of rare earth metals, with almost the same abundance as Ni, Cu, and Zn in the Earth’s crust, making up around 0.0046 wt% of the crust of the Earth [36]. The exploration of RE elements in combination with transition metals goes back to the 1970s, with the first synthesis of LaNi_5,_ used for water electrolysis, and the demonstration of the electrocatalytic hydrogen evolution activity [37] from the RE-TM and noble metals including Pt, Au, and Pd, gaining considerable interest in 21st century. Among several RE metals, Ce is by far the most explored element for electrocatalytic applications due to its superior redox capability, i.e., interconversion of Ce^4+^ ↔ Ce^3+^. Cerium, both in the form of nanoparticles and in an atomically dispersed state, is explored as an ORR electrocatalyst. The fluorite crystal structure of the CeO_2_ nanocrystal is with the space group *Fm3m* and the cell parameters are 0.5411 nm (a=b=c). In the CeO_2_ unit cell, each Ce^4+^ is coordinated with eight adjacent O^2−^ to form an octahedral interstitial, and each O^2−^ is coordinated with four adjacent Ce^4+^ to form a tetrahedral unit [38]. Because of its high electrical energy levels and unoccupied 4*f* orbital, CeO_2_ shows significant promise as a catalytic agent. The remarkable physical and chemical properties of Ce are mostly derived from its unusual electronic configuration, which is [Xe]4f^1^5d^1^6s^2^. Electrocatalytic activity can be enhanced by using Ce, a naturally occurring occupier of 4f orbital electrons, as an electronic modulator to build charge transfer highways close to the Fermi level [39]. Furthermore, Ce’s electronic properties and large atomic radius cause it to frequently display a highly coordinated structure with a minimum of four ligands, and its electronic configuration allows for a flexible variation in the coordination number [40]. This results in Ce-based materials having a highly tunable local coordination/geometry. Ce is a dopant that can be used to regulate the adsorption/desorption of chemical intermediates for electrocatalytic reactions, thanks to its high atomic radius [41]. In addition, the multivalence feature of CeO_2_ provides the potential to create robust interactions synergistically with other catalysts, hence improving the efficacy of electrocatalysis. The distinctive crystal structure and reversible valence properties of CeO_2_ facilitate the creation of oxygen vacancies, resulting in a defect-rich architecture that underpins its superior catalytic activity.

The oxygen vacancy, resulting from the reduction of Ce^4+^ to Ce^3+^ or the migration of lattice oxygen, significantly affects the electrical and chemical characteristics of CeO_2_. In catalysts, oxygen vacancies not only stabilize active component nanoparticles or clusters but also modulate the electrical structure of the catalysts and provide a faster charge transfer rate and excellent reaction kinetics [42,43]. During ORR electrocatalysis, the 4*f* band of Ce can hybridize with a 2*p* band of O_2_ intermediates because the oxygen-defective CeO_2_ lowers the 4f energy level of Ce below the Fermi level [44]. As a result, CeO_2_ shows promising results as an electrocatalytic promoter for improving the electrocatalytic activity of adjacent TM-based active sites [45,46]. Despite the several advantages of CeO_2_, poor electrical conductivity hinders its direct use as an electrocatalyst; therefore, the CeO_2_ nanoparticles, in general, require conducting carbon support to improve the interfacial electron transfer and to provide a platform to host the CeO_2_ nanoparticles. The combination of the high conductivity of rGO and the ample active sites on CeO_2_ renders the nanocomposite an effective electrocatalyst for ORR [47]. In addition, in several studies, it is found that the noble metal nanoparticles/CeO_2_/carbon support heterojunctions create a unique electrocatalyst in which CeO_2_ found to improve the catalyst activity and stability [48]. Not only are CeO2 nanoparticles known to improve electrocatalyst stability, but they are also known to improve the nafion membrane stability by averting membrane degradation. It is well known that during ORR, the electrocatalysts produce a considerable amount of H_2_O_2_ and OOH^−^, which is produced by a 2+2 O_2_ reduction pathway, detrimental to the nafion membrane and even the catalyst layer. CeO_2_ is the best known OH^·^ radical scavenging agent for fuel cells that include both membrane and catalysts. Therefore, plenty of researchers have introduced CeO_2_ nanoparticles incorporated into the membranes, aiming to improve stability [49,50].

Platinum group metals supported on a carbon matrix are the most popular catalysts for heterogeneous catalysts. As stated earlier, RE metals in the form of nanoparticles of metallic and metal oxides are often used as co-catalysts/promoters in combination with transition and noble metals [51]. However, when either catalysts, or co-catalysts with heterogenous catalysis, are used, only the surface atoms participate in the reaction, with most reactants never reach atoms in the bulk, therefore rendering them surplus [52]. Metal nanoparticles, for example, often have more than 40 atoms and have a diameter of about 1 nm. Despite environmental and chemical reaction-induced changes to the geometric configurations of exposed surface atoms (facets, corners, edges, metal–support interfaces, etc.), their geometric structures remain relatively un-stable and are thus less vulnerable [53]. In addition, the specific electronic structure of the metal–oxide interface in supported catalysts is crucial to the catalytic process since it can influence the selectivity of the products, the absorption and activation of reactants/intermediates, and the catalysts themselves [54]. Hence, a lot of work had gone into finding ways to make active metals more efficiently and to make supporting metal catalysts work better by making the active metals smaller [55]. The most efficient use of precious metals would be possible with nanoparticles that were smaller; this would be especially true for metals that were disseminated in the form of single atoms [56]. Since then, catalysis at single-atom sites has been expanding rapidly. Single-atom catalysts (SACs) illustrate the smallest dimension of metal catalysts, which not only enhances the atomic efficiency of supported metals but also boosts the quantity of interfacial sites; in contrast, the unexposed inner atoms of conventional nano-sized catalysts cannot interact with the support at interfacial sites [57]. Accordingly, several studies have been conducted in which SACs of RE metals have been proposed for ORR and other energy conversion and storage electrochemical reactions [51].

**Figure 2:**
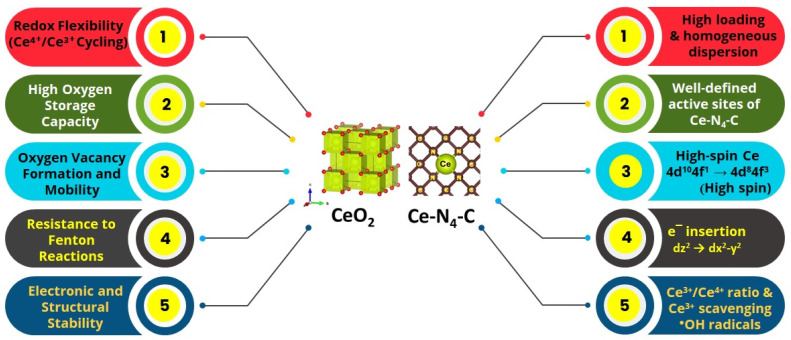
Advantages of CeO_2_ and Ce-N_4_-C for ORR.

The rare earth metal Ce is special among the several rare earth metals with regard to electrocatalysis, especially for ORR, as shown in Figure 2. The special features of Ce include (i) the redox couple of Ce^3+^/Ce^4+^ which accompanies the oxygen vacancy and ^●^OH radical scavenging, whereas most other RE elements largely exists in stable RE^+3^ oxidation states and lack the dynamic redox coupling ability; (ii) the unique fluorite structure of CeO_2_ which can host O_2_ vacancies without any lattice distortions which act as active sites for O_2_ adsorption, while most other rare earth oxides, for instance, La_2_O_3_ and Y_2_O_3_ with their rigid hexagonal structures, offer less oxygen vacancies and O_2_ storage capacity; (iii) the radical scavenging capacity of Ce, whereas other rare earths are known to lack radical scavenging capacity; (iv) the structure stability of CeO_2_, owing to its partially filled 4f orbitals; and (v) its ability to change the high-spin state in the atomically dispersed state, a unique property of Ce. These factors stand out as important aspects of Ce for ORR (Figure 2)

## 3. Pt/CeO_2_ Catalysts for ORR

It is well accepted that various metal oxides, including RE oxides, can induce a positive effect on Pt NPs, thereby enhancing the ORR process due to restructuring of Pt surface defects induced by the RE oxides [58]. In addition, the presence of Ce^3+^ was also found to enhance the metallic ability of Pt nanoparticles and effectively inhibit the oxidation of Pt nanoparticles [59]. In this regard, CeO_2_ as a metal oxide component presence in Pt-based catalysts could bring several advantages such as Pt/CeO_2_/C heterojunctions; the Pt nanoparticles’ binding strength at the CeO_2_ phase was found to enhance the stability of the catalyst in strong metal support interactions (SMSI). CeO_2_ can donate electrons to Pt and influence its electronic structure through electronic effects and oxygen absorption energy. Pt NPs can also be stabilized by CeO_2_ anchoring. CeO_2_ acts as a co-catalyst in Pt-CeO_x_/C catalysts, helping the main Pt catalytic center electronically and preventing Pt oxide production at high potentials through the substitutional Ce^3+^ to Ce^4+^ redox reaction and oxygen buffering. Pt-CeO_x_/C triple-phase hetero-interface structures improve ORR activity and fuel cell performance.

**Figure 3:**
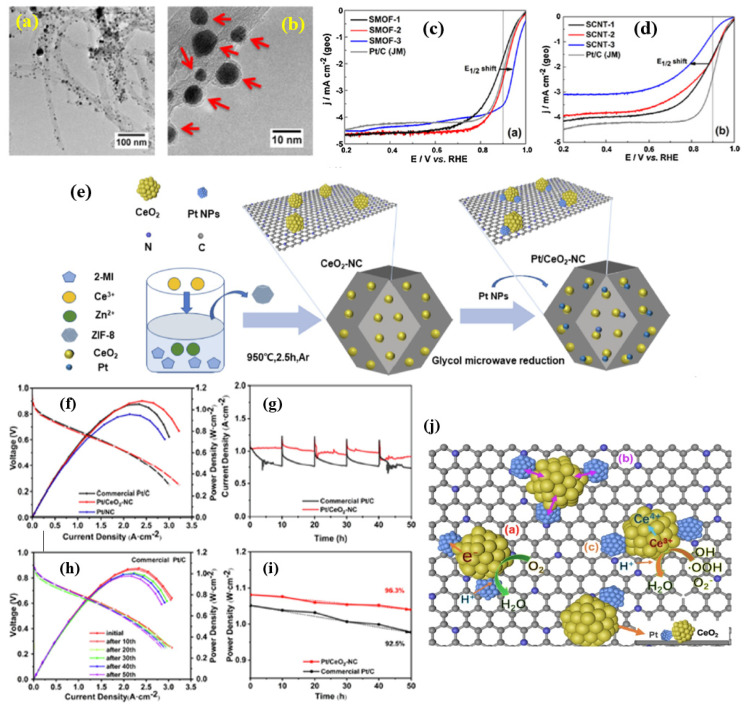
TEM images of SMOF-3 (**a**,**b**) and LSVs curves (**c**,**d**) of various SMOF catalysts at 900 rpm (Reproduced with permissions from Ref. [60]). (**e**) The schematic representation of Pt/CeO_2_-NC catalyst synthesis. Polarization curves (**f**), durability (**g**), and polarization curves recorded at specific time intervals (**h**), power density retention curves of Pt/CeO_2_-NC and Pt/C catalysts (**i**). (**j**) Diagram representing the role of CeO_2_ (Reproduced with permissions from Ref. [62]).

In this regard, in several studies, CeO_x_-interfaced Pt catalysts have been proposed as durable ORR catalysts. One such notable work is carried out by Luo et al. [60], who synthesized an advanced Pt/CeO_x_/C nanocomposite with porous carbon with multiwalled carbon nanotubes (MWCNTs) by utilizing Ce-incorporated MOFs synthesized with an ATPT = 2-aminoteraphthalate organic ligand. After a high temperature treatment, the MOF(Ce) transformed into Ce-O_x_, resulting in the formation of an oxide/carbon composite. The TEM images show that Pt nanoparticles of nearly spherical shapes, together with some amorphous carbon mixed oxide, are found to be present in an intimate, interconnecting way with Pt, Ce, O and C, forming a nanocomposite system. TEM images also clearly show the formation of fragments of Ce oxides in close connection with Pt nanoparticles (Figure 3a,b). The ORR analysis shows proportionately increased half-wave potential and improved Tafel slopes indicate that the formation of hetero-junctions of Pt NPs and CeO_x_/C helps in enhancing the ORR activity in acidic medium (Figure 3c,d). The mass and specific activities of the Pt/CeO_x_/C catalyst are shown to be 10 times greater than the commercial Pt/C catalyst. Metal oxides such as TiO_2_, SnO_2_, and WO_3_, including CeO_2_, have been used as potential carbon composites structures aiming for improved metal–metal oxide support interactions and improved corrosion resistance catalysts in fuel cells and metal–air batteries [61]. The triple-phase interface structures formed from the Pt, CeO_x_, and C, are known to be responsible for the strong metal–support interactions (SMSI). Several studies have concluded that the presence of SMSI is responsible for optimized ORR activity and stability of the catalysts. Among several metal oxides, CeO_2_ is the one most investigated as an active component of triple-phase interface structures, due to its defective crystal structure and rapid interchangeable oxidation states, from Ce^3+^ to Ce^4+^ and vice versa. In addition to this, CeO_2_ has excellent oxygen storage capacity, meaning that it has the ability to release O_2_ in environments with low O_2_ levels by absorbing O_2_ atoms from NO, H_2_O, and O_2_. CeO_2_ also performs excellently in scavenging hydroxyl (HO⋅) and hydroperoxyl (HOO⋅) radicals formed during the ORR process and mitigates the chemical degradation of the membrane and catalyst. Such triple-phase hetero-structures are constructed by packing nanosized CeO_2_ with ZIF-8-derived NC as a novel composite, supported in a study by Zhao et al. [62]. An abundant triple-phase hetero-junction of a Pt/CeO_2_-NC catalyst is created after Pt nanoparticles are deposited (Figure 3e). The Pt NPs are the ORR’s primary catalytic centers, and NC makes it possible for the Pt NPs to be evenly distributed and guarantees enough electrical conductivity. By donating electrons to Pt and influencing its electronic structure through electronic effects and oxygen absorption energy, CeO_2_ can play the role of an electron donor. Additionally, the anchoring effect between Pt and CeO_2_ can stabilize the Pt NPs. The catalyst made of 20% Pt/CeO_2_-NC exhibits an E_1/2_ of 0.922 V, and no loss after 10,000 cycles of ADTs in acidic media, according to RRDE tests, which is better than the commercial Pt/C. This is because of the interactions between Pt NPs, CeO_2_, and NC. Furthermore, when comparing 20% commercial Pt/C to 20% Pt/CeO_2_-NC in PEMFC single-cell measurements, the latter exhibits superior electrochemical activity and durability, even at lower cathode Pt loadings. To construct the effective triple-phase hetero-junctions, first, Ce@ZIF-8 structures are synthesized from 2-MIM, Ce^3+^, and Zn^2+^ structures in methanol solution which are then pyrolyzed under N_2_ atmosphere, which results in the formation of CeO_2_ nanoclusters on to which Pt nanoparticles are deposited using a microwave polyol method.

TEM images clearly reveal the formation of abundant three-phase (i.e., Pt NPs, CeO_2_, and NC) interfacial structures in Pt/CeO_2_-NC catalyst. It is seen that the CeO_2_ (111) phase is in close proximity to the Pt (111) NPs, suggesting an efficient catalyst with abundant triple-phase interfacial structures. The strong anchoring effect between Pt NPs and CeO_2_-NC is responsible for the more uniform distribution of Pt NPs on CeO_2_-NC supports compared to NC. The strong SMSI effect is further revealed by the XPS analysis, in which there is a shift in the Pt 4f peaks which is ascribed to the strong interactions between CeO_2_ and Pt. The ORR activity of the Pt/CeO_2_-NC catalyst showed excellent kinetics, with obtained half-wave potentials of 0.922 V, much higher than those of Pt/C, Pt-NC, and Pt/CeO_2_; also, the highest ECSA of Pt/CeO_2_-NC is 86.8 m^2^⋅g _Pt_^−1^, which is the largest one among these catalysts. In addition to the ORR activity, it was found that Pt/CeO_2_-NC exhibited excellent stability, with a loss of just 10 mV in its half-wave potential after 10,000 ADT cycles, in contrast to 35 mV loss in half-wave potential for Pt/C, with a proportional loss of ECSA of 26%, whereas the loss in ECSA is just 9% for Pt/CeO_2_-NC catalyst. Because of this, Pt/CeO_2_-NC outlasts commercial 20% Pt/C in terms of durability. This is because CeO_2_-NC has an anchoring effect on Pt NPs, which forbids their growth and aggregation. Interestingly, when applied as a cathode catalyst in PEMFC fuel cell configuration, the Pt/CeO_2_-NC catalyst delivered a power density of 1.08 W cm^−2^, higher than the Pt/NC and Pt/C catalysts (Figure 3f–j). The enhanced ORR activity, stability, and fuel cell performance of the Pt/CeO_2_-NC catalyst was attributed to several causes, among which “an effective three phase interface structure” was the main one [63]. By influencing the electronic structure of Pt and the energy of oxygen absorbed on Pt sites, CeO_2_ can increase the activity of the ORR. Pt nanoparticles are uniformly anchored by the CeO_2_-NC supports because of the anchoring effect [64,65]. Because the anchoring effect prevents Pt NPs from growing into aggregates, they enhance the stability of the catalysts [66,67]. The free radical scavenger properties of CeO_2_ nanoparticles in PEMFCs are well-known. Ce^3+^ and Ce^4+^ are present in CeO_2_ due to the presence of oxygen vacancies in the material. The antioxidant properties of CeO_2_ prevent the breakdown of the nafion membrane and catalyst support, which prolongs the life of Pt/CeO_2_-NC in PEMFCs [68].

**Figure 4:**
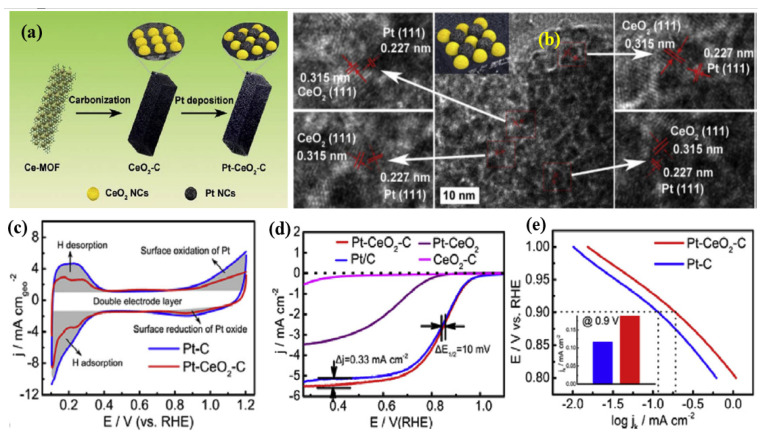
Schematic representation (**a**). HR-TEM images (**b**). CV curves recorded in N_2_ atmosphere in 0.1 M HClO_4_ electrolyte (**c**). LSV curves recorded in O_2_ atmosphere in 0.1 M HClO_4_ electrolyte at 1600 rom (**d**,**e**) and Tafel curves of Pt-CeO_2_-C catalysts at 0.9 V (Reproduced with permissions from Ref. [73]).

Alloying Pt with other metals and metal oxides is known to down-shift the *d*-band center of Pt; as a result, the ORR intermediates absorb optimally on the active sites [69]. However, the alloyed element or metal oxide leaches out gradually, resulting in long-term instability of the electrocatalyst [70]. The mitigation of the effects of the alloying element and metal oxide component on the electrocatalyst is highly desired to improve the electrocatalytic performance of Pt for ORR. It is well known that CeO_2_ can enhance carbon corrosion resistance in acid due to its strong electron interaction with Pt and its high stability in acidic environments [71,72]. Nevertheless, the use of Pt-CeO_2_ binary catalysts in ORR has been greatly hindered by CeO_2_’s poor electronic conductivity. An effective strategy to improve electronic conductivity while maintaining the strong interaction between Pt and CeO_2_ is to construct a Pt-CeO_2_/C ternary nanostructure by introducing carbon into the hybrid catalyst. Du et al. [73] fabricated a Pt/CeO_2_-C ternary mutual interacted nanostructure from a crystal feature of Ce-MOF, resulting from the thermal treatment of Ce-MOFs, resulting in the formation of abundant tiny CeO_2_ nanoclusters (~2 nm) on to which Pt nanoparticles were deposited (Figure 4a). The electronic conductivity, ORR catalytic activity, and long-term durability of ORR are all greatly enhanced by the closely packed structure, which can form enough interfaces for electronic interaction and electron transfer. HRTEM images suggest that CeO_2_ nanocrystals are well-structured, with a cluster size of 2 nm, and CeO_2_ NCs produced by Ce-MOF avoided aggregation, which may be explained by the unique backbone properties of Ce [74] that cause the nanorod morphology of CeO_2_/C (Figure 4b). XPS analysis reveals that the Ce^3+^ molar ratio in CeO_2_/C and Pt/CeO_2_/C catalysts were found to be 14.3% and 20.5%, respectively. It is possible that the partial reduction of Ce^4+^ during Pt deposition is responsible for the increase in the Ce^3+^ component, and that Ce^3+^ acts as a reducing agent to create small Pt NCs embedded on the surface of CeO_2_ in an alkaline environment. A prior study using in situ electrochemical X-ray absorption fine structure demonstrated that the small amount of Ce^3+^ in CeO_2_ hinders the production of Pt oxide at high potentials through the substitutional oxidation of Ce^3+^ to Ce^4+^. This has positive implications for enhancing the catalytic performance of Pt in ORR [59,75]. The ORR studies suggest that the Pt/CeO_2_/C catalyst showed excellent ORR activity, with its kinetics equaling commercial Pt/C catalysts, which is attributed to the enhanced charge associated with H_2_ adsorption/desorption, with nearly 4 electron transfer of O_2_ reduction (Figure 4c–e). The enhanced Pt stability may be due to the confinement of Pt NCs in the CeO_2_/C hybrid and the synergistic effect between the NCs and the CeO_2_ support, specifically the electron deficiency of small Pt NCs caused by the transfer of electrons from Pt to CeO_2_. The decrease in the Pt-Pt bond distance because of the Pt-O interaction may be responsible for the enhancement of ORR activity [76].

In addition to their dual role as co-catalysts and supports, metal oxides like CeO_2_ can modify the electronic structure of Pt NPs and thereby produce a synergistic effect with Pt. Among the oxygen catalytic reactivity compounds, CeO_2_ stands out due to its superior chemical stability and noticeably reduced cost. On the other hand, a carbon layer may be necessary to enhance the anchoring effect of Pt on CeO_2_ due to its reduced electrical conductivity. Wang et al. [77] developed a “Pt-oxide”-based composite electrocatalyst of “CeO_2_ overlapped with nitrogen-doped carbon layer anchoring Pt nanoparticles” (Pt-CeO_2_@CN). The TEM measurements make it very clear that the catalyst contains Pt NPs, CeO_2_, and N-doped carbon. Specifically, it shows that the CeO_2_ layer overlaps with the N-doped carbon layer, which makes Pt NPs well-anchored. Furthermore, the HR-TEM analysis also clearly reveals that the distinct crystal plane distances are 0.223 nm for the Pt (1 1 1) plane and 0.311 nm for the CeO_2_ (1 1 1) plane, respectively. XPS analysis reveals a high abundance of graphitic-N and pyridinic-N species. Pt 4f spectra further confirm the anchoring effect of Pt on the CeO_2_ interfaced by N-doped carbon, which enhances the electron transport from the support to the Pt catalytic active sites. The ORR activity of the Pt-CeO_2_@CN is found to be higher than the commercial Pt/C catalyst. The onset potential and half-wave potential of the Pt-CeO_2_@CN catalyst is superior by 16 and 29 mV, respectively, when compared to Pt/C catalyst, suggesting that CeO_2_ and N-doped carbon helps to enhance the catalytic activity.

In a similar trend, Chen et al. [78] developed an atomic layer deposition-produced Pt/CeO_2_/CNT triple-junction interface, showing improved ORR activity and durability. SMSI have been proven to not only to enhance the strength of supported metal nanoparticles but also to help in the dispersion of Pt nanoparticles. In addition, the SMSI helped to prevent Pt detachment and further aggregation during long-term potential cycling, therefore helping to enhance the catalyst stability. Kinetically, the SMSI also promote the absorption of oxygen and cleavage of the O=O bond on the Pt surface [79]. Although Pt-MO_x_-C triple junctions are typically produced by wet chemical synthesis, it is challenging to precisely control the catalyst’s nucleation and growth during the reaction, leading to potentially larger size and uneven distribution. On the other hand, atomic layer deposition is a great way to make nano catalysts, thin films, and metal and metallic compound catalysts with clusters, single atoms, and highly dispersed nanoparticles. In this work, clusters of CeO_2_-nanoparticles were deposited on a CNT which is then used as a substrate on to which Pt nanoparticles are deposited using the ALD method. It was found that the Pt/CeO_2_/CNT-A triple-junction catalyst showed enhanced ORR activity compared to the Pt/CeO_2_/CNT-W that is synthesized by a wet chemical synthesis method. The XRD analysis reveals that Pt nanoparticles deposited by ALD show smaller crystallite size, with an average size of 8.30 nm, whereas, for wet chemically synthesized catalysts, it is 10 nm. TEM images clearly show that the Pt NPs were deposited on the of CeO_2_ and no visible CNTs were seen with Pt NPs, suggesting that most of the Pt NPs were deposited on the CeO_2_/CNT, confirming that effective Pt/CeO_2_/CNT-A triple-junction structures were generated. Furthermore, the XPS analysis revealed that the Pt/CeO_2_/CNT-A catalyst contains abundant oxygen vacancies (V_o_), which are essential for the interaction between Pt and CeO_2._ The XPS spectra also reveal that Pt/CeO_2_/CNT-A contains high amount of Ce^3+^, resulting from the electron transfer from Pt to the CeO_2_ due to the reduction in Ce^4+^ ions [80]. Pt 4f spectra of Pt/CeO_2_/CNT-A and Pt/CeO_2_/CNT-W also suggest a clear differentiation between the Pt nanoparticle deposition method-induced ORR activity. A negative shift of 0.14 eV is observed for Pt/CeO_2_/CNT-A compared with Pt/CeO_2_/CNT-W. According to the *d*-band theory, negative shift in the binding energy indicates stronger electronic interaction, which is consistent with the observed increase in Ce^3+^ content. The negative shift in binding energy also means the lowering the *d*-band center of Pt NPS, which further helps in enhancing the electrocatalytic activity [81]. The ORR studies indicate that both Pt/CeO_2_/CNT-A and Pt/CeO_2_/CNT-W catalysts show enhanced ORR activity, with 25 and 10 mV-higher half-wave potential representing the paramount interfacial structures of CeO_2_ and CNT in enhancing the ORR activity. Furthermore, electron transfer from the Ce^3+^ to Ce^4+^ oxidation state further strengthens the nucleation and Pt-CeO_2_ interface interaction. In addition, the high V_o_ and oxygen storage capacity of CeO_2_ promotes the optimal adsorption of oxygen and cleavage of the O=O bond in the Pt surface [82]. In addition to the excellent ORR activity, Pt/CeO_2_/CNT-A also showed excellent stability.

To briefly conclude, it is found that Pt-CeO_x_/C triple-phase hetero-interface structures with Pt enhanced the binding strength of Pt nanoparticles with the Pt-CeO_x_/C junctions, which results in SMSI which is responsible for the enhanced ORR activity and stability of the catalysts. By donating electrons to Pt and influencing its electronic structure through electronic effects and oxygen absorption energy, CeO_2_ can play the role of an electron donor. Additionally, the anchoring effect between Pt and CeO_2_ can stabilize the Pt NPs. In Pt-CeO_x_/C catalysts, CeO_2_ plays the role of co-catalyst, assisting in the main Pt catalytic center electronically, and hinders the production of Pt oxide at high potentials through the substitutional Ce^3+^ to Ce^4+^ redox reaction and CeO_2_ oxygen buffering capacity. The presence of Pt-CeO_x_/C triple-phase hetero-interface structures not only enhances the ORR activity but also influences the fuel cell performance.

## 4. Fe/CeO_2_/C Catalysts for ORR

CeO_2_ is an important type of metal oxide, useful for various electrochemical reactions including CO oxidation, NO reduction, and for ORR, due to its unique oxygen storage capacity and facile interconversion of Ce^3+^ to Ce^4+^, in addition to its superior property of corrosion resistance and optimal absorption of reaction species on to its surface [26]. Ce is found to regulate Fe’s electronic structure and spatially confines and stabilizes Fe atoms. O_2_ interaction on Fe active sites is enhanced by 4f^1^ (Ce^3+^) localized electron transfer to d-orbitals due to differences in electronegativity values between Ce and Fe. Transition metal atoms like Fe interact well with Ce 4f^1^ of (Ce^3+^). Hybridizing 4f-3d orbitals lowers the band gap and improves conduction band dispersion, boosting ORR. Thus, the catalyst must have a high Ce^3+^ ratio. Hybridizing d-f orbitals reduces the Fe atom’s d-band center, making electron transfer to adsorbed OH intermediates easier and speeding up the rate-determining step and ORR. Thus, in Fe-Ce dual-atom-based catalysts, Fe is the main active center and rare earth Ce 4f electrons improve charge transfer, reduce the d-band center, and improve ORR kinetics. In this section, we will describe the various Fe/Ce-based catalysts proposed in the literature.

**Figure 5:**
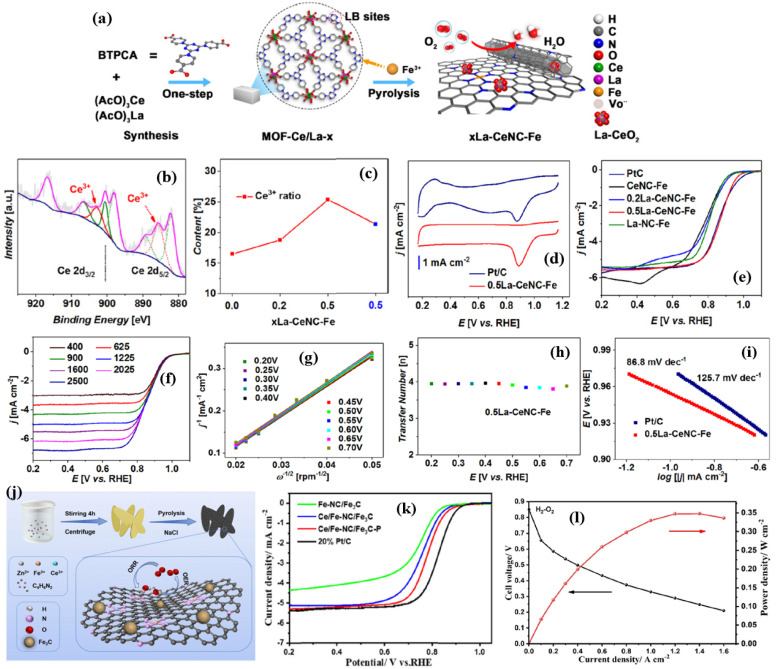
(**a**) Schematic representation of 0.5La-CeNC-Fe. (**b**) XPS deconvoluted peaks of Ce^3+^ and (**c**) obtained Ce^3+^ content. (**d**) CV and (**e**) LSV curves of various 0.5La-CeNC-Fe catalysts. (**f**) LSVs recorded at various rotations per minute. (**g**) K–L plots. (**h**) Number of electrons. (**i**) Tafel plots of 0.5La-CeNC-Fe catalysts (Reproduced with permissions from Ref. [83]). (**j**) Schematic synthesis of Ce/Fe-NC/Fe_3_C-P catalyst. (**k**) LSV curves of various Ce/Fe-NC/Fe_3_C-P catalysts in 0.1 M HClO_4_. (**l**) PEMFC polarization curves of Ce/Fe-NC/Fe_3_C-P catalyst (Reproduced with permissions from Ref. [85]).

Zhang et al. [83] synthesized a series of MOFs with Ce/La MOFs and investigated the effect of Lewis’s base sites, composed of rare earth metals, on the synergistic ORR activity of porous Fe-Nx active sites (Figure 5a). The Le/Ce-doped MOFs were synthesized by a simple co-precipitation method, and then the synthesized MOFs were immersed in the Fe^3+^ solution. The resulting Fe-MOFs were then pyrolyzed to obtain the xLa-CeNC-Fe. Analysis of the diffraction peaks of Ce-NC-Fe with La^3+^ show that the diffraction peaks shift to a lower 2theta angle, indicating the successful incorporation of La^3+^ (0.116 nm), whose ionic radius is larger than the Ce^4+^ (0.097 nm). Both SEM and TEM images show CNT structures, obviously due to the ability of Fe to graphitize the carbon during the pyrolysis process. The ORR analysis of the 0.5La-CeNC-Fe catalyst showed a well-defined cathodic reduction peak in the cyclic voltametric analysis, with a peak potential of 0.886 V vs. RHE, much higher than the Pt/C catalyst of 0.875 V. The catalyst of 0.5La-CeNC-Fe with an E_1/2_ of 0.870 V exhibits significantly better ORR activity than that of other catalysts and is slightly better than that of Pt/C with an E_1/2_ of 0.862 V. The K-L plots indicate that the 0.5La-CeNC-Fe catalyst exhibited an ‘n’ of 3.94, indicating almost a direct four-electron reduction of O_2_ to H_2_O (Figure 5b–i).

In addition to the role of Ce as a co-catalyst, O_2_ buffering agent, Ce plays an important role in spatially confining and stabilizing Fe atoms [84]. Thus, Ce can greatly improve the ORR/OER catalyst’s stability and catalytic activity when added to metal-based hybrid electrocatalysts. Wang et al. [85] synthesized a two-dimensional ZIF assisted with a NaCl molten salt synthesis method to create a Ce/Fe-NC/Fe_3_C-P nanosheet with a hierarchical porous structure (Figure 5j). Thanks to the abundance of molten salt around the ZIF nanosheets, the precursor’s shape was kept intact. At the same time, adding molten salt of sodium chloride to a carbon matrix can improve electron transport, increase the catalyst’s durability, and raise the degree of local graphitization and graphitic N dopants. Both Fe-ZIF and Ce/Fe-ZIF exhibit an elliptical nanoflake structure, and the Fe-ZIF-derived Fe-NC/Fe_3_C catalyst exhibited structures that resembled carbon nanotubes (CNTs), with a significant amount of agglomeration. On the other hand, the Ce-ZIF-pyrolyzed Ce/Fe-N-C/Fe_3_C-P catalyst displayed a very small number of CNT-like structures. This is because the addition of Ce causes the Ce/Fe-ZIF nanoflakes to shrink and link with each other during the pyrolysis process, resulting in the formation of a coralloid structure. According to this, Ce is responsible for regulating the electronic structure of Fe and preventing the growth of carbon nanotubes. Furthermore, it was found that the ratio of molten salt NaCl:Ce/Fe-ZIF also plays a role in preserving the elliptical nanoflake structures. Obtaining nanosheets with a mass ratio of 1:1 between NaCl and Ce/Fe-ZIF results in significant agglomeration. The elliptical nanoflake structure of Ce/Fe-ZIF was integrally preserved when the ratio increased to 3:1. As the ratio increased to 5:1, the morphology became nearly identical to that of 3:1. HR-TEM images of the Ce/Fe-NC/Fe_3_C-P catalyst show the homogeneous distribution of C, Fe, and Ce, along with the Fe_3_C nanoparticles, meaning that Fe particles are surrounded by the N-doped carbon shells which act as a catalytic active site to facilitate the ORR/OER [86,87,88,89,90]. The core–shell structure is crucial for avoiding NP agglomeration and boosts catalyst durability by halting Fe_3_C nanoparticle leaching in extreme electrochemical environments. Interestingly, it is also found that the Ce also regulates the porosity of the catalysts. According to the pore size distribution analysis, micropores are more prevalent in Fe-NC/Fe_3_C, but mesopores appear after Ce incorporation in the Ce/Fe-NC/Fe_3_C-P catalyst. This could be because the preexisting Ce controlled the electrical structure of Fe and prevented the growth of CNTs, in contrast to Fe-NC/Fe_3_C [91]. In addition, when NaCl was introduced during the synthesis process, it was found that well-balanced micro and mesopores were identified in the catalyst [92]. The role of micro and mesopores includes hosting the edge, highly active ORR sites in the form of Fe-N_x_-C and providing a pathway for the reactants and products to the ORR active sites, respectively [93]. The XPS analysis reveals the presence of definite Ce-N_x_ bonding active sites along with pyridinic-N, Fe−N*_x_*, pyrrolic-N, and graphitic-N, which can act as ORR active sites. There is a noticeable change in the binding energy of Fe2p_3/2_ between the Fe-NC/Fe_3_C and Ce/Fe-NC/Fe_3_C-P catalysts, which is an intriguing finding. This might be because Ce doping allows charges to migrate between Fe and N elements, among others. The charge transfer effect, according to earlier research, lowers the *d*-band center value, which in turn reduces the adsorption energy of oxygen-containing intermediates in electrochemical reactions and increases the ORR electrocatalytic efficiency [94]. The electrochemical ORR studies indicate that the Ce/Fe-NC/Fe_3_C-P catalyst showed the highest ORR activity, followed by Ce/Fe-NC/Fe_3_C, 20% Pt/C, and Fe-NC/Fe_3_C. The Ce/Fe-NC/Fe_3_C-P catalyst’s outstanding performance in ORR is likely connected to Ce species, which effectively lower the energy barrier of the rate-limiting step and promote more efficient transport of oxygen-containing intermediates containing H_2_O_2_, OH^−^, and *OH radicals during the ORR. When applied as a cathode catalyst in Zn–air batteries and in PEMFC environments, the Ce/Fe-NC/Fe_3_C-P catalyst delivered a powder density of 184 and 347 mW cm^−2^, respectively (Figure 5k,l).

**Figure 6:**
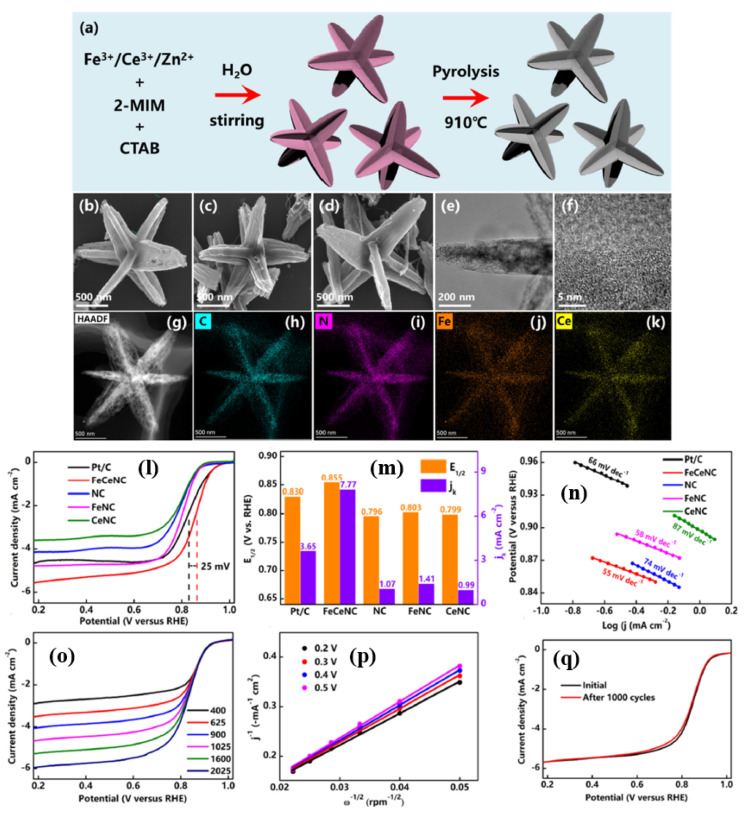
(**a**) Schematic representation of the synthesis process of the FeCeNC catalyst with CTAB as surfactant. (**b**–**g**) HR TEM images. (**h**–**k**) Elemental mapping for C, N, Fe, and Ce atoms in FeCeNC catalyst. (**l**) LSV curves. (**m**) Relationship between E_1/2_ and J_k_. (**n**) Tafel plots. (**o**) LSVs recorded at different rpms. (**p**) K–L plots. (**q**) Stability curves of FeCeNC catalysts (Reproduced with permissions from Ref. [95] open access).

In another study, Zenghui et al. [95] synthesized a unique three-dimensional (3D) star-like carbon material electrocatalyst made of Fe and Ce, synthesized by a ZIF-8 structures with Zn^2+^ and 2-MIM and the Fe^3+^ and Ce^3+^ ions then incorporated into the ZIF-8 structures in the presence of a CTAB surfactant, and pyrolyzed at 910 °C, followed by acid etching (Figure 6a). SEM and TEM measurements prove that the FeCeNC catalyst has a star shape, where each corner is of the same size and all corners are oriented in different directions in three dimensions. While the synthesis of the FeCeNC catalyst looks almost very similar to the simple, traditional ZIF-8 synthesis, the only difference is the use of the CTAB surfactant; therefore, the evolution of the 3D star-shaped FeCeNC catalyst could be primarily attributed to the use of CTAB (Figure 6b–k). The ORR activity of FeCeNC catalyst was evaluated in 0.1 M KOH solution. The order of ORR activity is as follows: NC < CeNC < FeNC < FeCeNC catalysts with a half-wave potential of 0.796, 0.799, 0.803, and 0.855 V vs. RHE, respectively. The ORR activity of the final and optimized FeCeNC catalyst is also higher than the commercial catalysts Pt/C by 25 mV. However, the number of electrons transferred per O_2_ molecule is slightly lower than the catalysts discussed so far, with an ‘n’ value of 3.7 derived from the K-L plots. Thanks to the strong bond between Fe, Ce, and the coordinating N atoms (Fe-N and Ce-N), FeCeNC also showed remarkable durability, with only a 2 mV decay after 1000 cycles. When assembled with FeCeNC as a cathode catalyst with rechargeable ZAB, it delivered a power density of 169.2 mW cm^−2^, much higher than the Pt/C catalyst which only delivered 88.0 mW cm^−2^. Furthermore, after 80 h of cycling, the charge–discharge efficiency of the ZAB battery dropped by just 6.8%, demonstrating excellent durability thanks to the higher power density and FeCeNC catalyst (Figure 6l–q).

In another study, Kumar et al. [96] synthesized highly efficient ZIF-derived Fe_x_Ce_y_@N–C composite catalysts for ORR. In this study, a simple ZIF-8 synthesis in the presence of Ce^3+^ and Fe^3+^ with ZIF-8 and MWCNTs, which were then pyrolyzed at 900 °C, generated highly defect-rich and disordered structured electrocatalysts of a Fe_x_Ce_y_@N–C (x:y =1:1) composition, which offer a greater tendency to adsorb O_2_ and promote the ORR kinetics which contains Fe-N_x_ and Ce-N_x_ active sites for ORR. In particular, it was well documented that Fe-N_x_ is the most accepted form of catalytic active site of M_x_-N_y_-C, and Ce-N_x_ takes a dual role as an ORR active site and a scavenger of free radical that are generated from Fe-Nx sites, as a result of the 2+2 electron reduction reaction, and mitigate catalyst degradation from reactive oxygen species (ROS) generated nearby the iron centers.

The XRD analysis of the catalysts reveals both metallic Fe and Ce and Fe-Ce alloy phases. XPS analysis reveals several important reasons contributing to the ORR activity of the Fe_x_Ce_y_@N–C catalyst, especially in Fe3p and Ce3d XPS spectra, as discussed below. The XPS peaks at 710.8 and 723.1 eV could potentially be indicative of the Fe^3+^ and Fe^2+^ species present in the octahedral places. The exclusive presence of divalent Fe is indicated by the satellite peak at approximately 716.0 eV. The adsorption of O_2_ intermediates is directly impacted by the movement of Fe in or out of the N_4_ plane, caused by changes in potential which correspond to changes in its oxidation state from +2 to +3. The structural distortion caused by substituting Fe^3+^ for Zn^2+^ in ZIF-8, as shown in experimental studies, is less stable because of the smaller ion radius (49–55 pm) [97]. Initial reduction of Fe^3+^ to Fe^2+^ in active sites improves ORR kinetics, which in turn helps to displace adsorbed H_2_O with O_2_ and facilitates electron transfer from Fe^2+^ to O_2_ [98,99]. Thus, by supplying evenly distributed Fe-N_x_ moieties and easing the adsorption of O_2_ intermediates, Fe^2+^ improves the ORR electrocatalytic activity. The Ce_3_d XPS spectra show the co-existence of Ce^3+^3d_3/2_ (3d^10^ 4f^1^) and Ce^4+^ (3d^10^4f^0^). In particular, the presence of Ce^3+^3d_3/2_ (3d^10^ 4f^1^) with the 4f^1^ orbital and a lone pair of electrons is an important aspect of the Ce-based catalysts. Ce 4f^1^ electrons a have high affinity to interact with transition metal atoms such as Fe and help in electron transfer from Ce → Fe, further enhancing the interaction of O_2_ on Fe active sites, due to the 4f^1^ localized electron transfer to d-orbitals of Fe active site due to their difference in their electronegativity values [100]. This hybridization of 4f-3d orbitals reduces the band gap and enhances the conduction band dispersion, facilitating efficient ORR. To briefly conclude, the Fe^2+^/^3+^ and Ce^3+^/^4+^ redox active couples promote enhanced ORR kinetics. As expected, a clear demonstration of the synergistic effects of Fe^2+^/^3+^ and Ce^3+^/^4+^ on the ORR activity has been established in the RDE studies in 0.1 M KOH solution. The ORR onset potential of 0.92 V is recorded for the Ce@NC catalyst, whereas the Fe1Ce1@NC catalyst ORR onset potential was found to be around 0.981 V, with a half-wave potential of 0.867 V vs. RHE, higher than that of 30 mV from the Pt/C catalyst.

**Figure 7:**
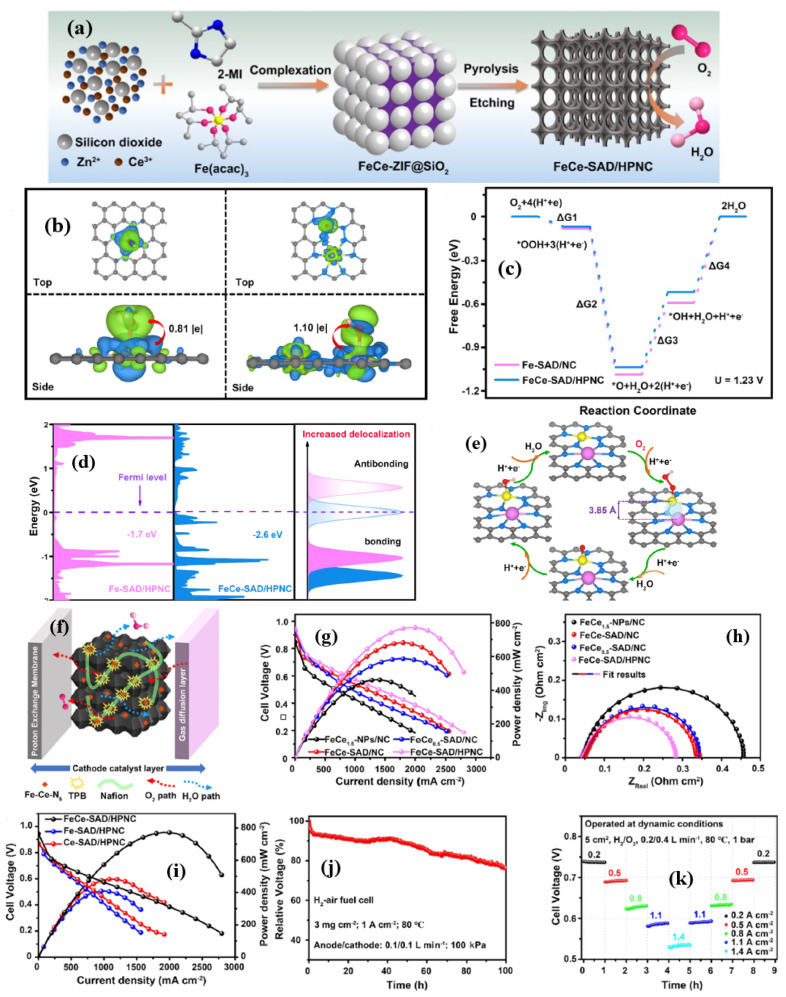
(**a**) Schematic of FeCe-SAD/HPNC synthesis. (**b**) Charge density. (**c**) Free energy diagram. (**d**) PDOS. (**e**) ORR pathway of FeCe-SAD/HPNC catalysts. (**f**) Typical representation of PEM cathode. (**g**) PEM polarization curves. (**h**) Impedance curves of various compositions of FeCe-SAD/HPNC catalysts. (**i**) PEM polarization curves of Fe-SAD/HPNC, Ce-SAD/HPNC, and FeCe-SAD/HPNC cathodes. (**j**) Stability curves at constant current and (**k**) various current density of FeCe-SAD/HPNC catalyst (Reproduced with permissions from Ref. [101]).

From the discovery of single-atom catalysts, research on embedding two or more atoms of different properties, creating dual-atom and multiple-atom catalysts, has become popular. Due to complexity of the multi-atom-based catalysts, dual-atom-based catalytic systems have recently attracted tremendous attention due to the easier gauge of the synergistic effects between the different metal atoms. Yang et al. [101] demonstrated the effect of Fe and Ce dual-metal catalysts (Fe-Ce-SAD/HPNC) to modulate the d-f orbital hybridization with an optimal O-binding energy to create an excellent ORR catalyst. It was found that, by hybridizing d-f orbitals, the d-band center of the Fe atom decreases, thus making the transfer of electrons to the adsorbed OH intermediates easier, therefore helping in accelerating the rate-determining step and thus the ORR (Figure 7a–e). The XANES study of the Fe-Ce-SAD/HPNC revealed that Fe atoms were positively charged, with Ce in the form of a valence between +3 and +4. Furthermore, the Ce L_3_-edge XANES spectrum suggests that Ce can exist in the form of isolated atoms, not bonded to Fe. However, the EXAFS spectrum indicates that Ce is bonded to -N due to Ce−N scattering, indicating that catalyst have atomically dispersed Ce-N sites. The ORR studies indicate that the Fe-Ce-SAD/HPNC catalyst showed a positive ORR onset potential of 0.91 V, a half-wave potential of 0.81 V, and a J_k_ of 32.7 mA cm^−2^ at 0.75 V vs. RHE.

In addition, the Fe-Ce-SAD/HPNC catalyst exhibited the highest double-layer capacitance of 53 mF cm^−2^ among all the catalysts in the study, indicating that the Fe-Ce-SAD/HPNC catalyst possess a higher surface area and hence high ORR activity. Furthermore, the reactive oxygen species (ROS) that include H_2_O^−^ or H_2_O_2_ and OH^●^ formed during the ORR process is one of the major issues that elevates the degradation of several components; therefore, it is important to quantify the ROS of the catalysts [102]. The qualitative and quantitative analysis of ROS is generally performed by the UV spectroscopy analysis with the help of 2,20-azinobis(3-ethylbenzthiazoline-6-sulfonate) (ABTS) as a substrate, that reacts with ROS formed during the ORR to form a green-colored solution upon reacting with ROS, with an absorption maxima at 417 nm. Interestingly, the Fe-Ce-SAD/HPNC catalysts showed lower intensity of the peak at 417 nm, along with the visual appearance of a light-colored solution, indicating that the Fe-Ce-SAD/HPNC catalyst forma less ROS during ORR. In addition to the RDE studies, the authors also evaluated the practical application of the Fe-Ce-SAD/HPNC catalyst in PEMFC conditions. The Fe-Ce-SAD/HPNC catalyst delivered a powder density of 0.771 W cm^−2^, with a catalyst loading of 3 mg cm^−2^, at a pressure of 1 bar H_2_-O_2_ and 0.498 W cm^−2^ in H_2_-O_2_ (Figure 7f–k).

The ORR mechanism of Fe-Ce-SAD/HPNC catalysts is assessed using density functional theory analysis by constructing a model of an active site composed of N_4_−Fe−Ce−N_6_−C and Fe−N_4_−C active sites for understanding the differences in the ORR mechanism [101]. The distance between Fe and Ce atoms was adjusted to 0.385 nm. The obtained Bader charge analysis reveals charge transfers of 0.81 and 1.10e^−^ for Fe-SAD HPNC and Fe-Ce-SAD/HPNC for the adsorbed *OH, confirming that the higher electron density around the Fe-Ce-SAD/HPNC catalyst is due to efficient electron transfer from Ce to Fe, enhancing the adsorption of O_2_. The density of states (DOS) further reveals that the d-band center of Fe in Fe-Ce-SAD/HPNC catalyst significantly decreased and that more occupied orbitals start to appear near the Fermi level due to *f-d* orbital interaction. The decreased *d*-band center means that more and more electrons are being added from Fe to the ORR intermediates such as -OH, which, therefore, reduces the adsorption strength between the ORR intermediate and the Fe-Ce-SAD/HPNC catalyst surface. The obtained *d*-band center from the DOS values relative to the Femi levels are −1.7 and −2.6 eV, respectively. The calculated free energy values were derived for various ORR intermediates such as *OOH, *O, and *OH. It is seen that there is a significant difference in the RDS of Fe-SAD HPNC and Fe-Ce-SAD/HPNC catalysts. For FeCe-SAD/HPNC catalysts, it is *O + H^+^ + e− → *OH, whereas for Fe-SAD/HPNC, it was *OH + H^+^ + e− → H_2_O, indicating that Ce 4F weakens the adsorption strength between the Fe active sites and the ORR intermediates, shifting the reaction mechanism with *OH desorption to *O > *OH. Therefore, it can be inferred that in Fe-Ce dual-atom-based catalysts, Fe is the main active center and rare earth Ce 4f electrons help to enhance the charge transfer and reduce the *d*-band center and hence enhance the ORR kinetics.

In a study, it was found that, in the process of Pt anchoring onto CeO_2_ through a high temperature treatment, the Pt atoms are tightly trapped on the ceria surface. Rather than diffusing int to the bulk phase of ceria and DFT, studies further indicate the stabilization effect of ceria for various metals such as Ni/Pd/Pt; essentially, CeO_2_ acts as dispersing agent. Based on these assumptions, Li et al. [103] explored the possibilities of stabilizing atomically dispersed Fe via ceria confining and trapping. With the CeO_2_ confining and trapping strategy, an Fe content as high as 4.6 wt% has been achieved. The Ce^3+^ and Fe^3+^ were adsorbed on the polypyrrole nanowires through electrostatic attraction. The metal ions are attached to the polypyrrole via backbone N atoms and the resulting material is then thermally treated to synthesize Ce/Fe-NCNW. Among various compositions of Ce^3+^ and Fe^3+^, the atomic ratio of 1:1 Ce^3+^ and Fe^3+^ is found to be optimal in terms of ORR activity. The Ce/Fe-NCNW catalyst showed an excellent half-wave potential of 0.915 V, higher than Pt/C by 49 mV, and a 3.3-times higher kinetic current density than Pt/C in 0.1 M KOH electrolyte, with a HO_2_^−^ yield of 2.5%. In a fuel cell comprising an alkaline membrane, the Ce/Fe-NCNW catalyst delivered a power density of 496 mW cm^−2^, with a catalyst loading of 1.0 mg cm^−2^ at 30 pis pressure. The catalyst synthesized without Ce showed lower ORR activity, confirming the definite role of ceria in ORR catalysis. The structural analysis of the Ce/Fe-NCNW catalyst indicates no visible agglomeration of Fe, whereas nanoparticles of CeO_2_ were observed. A high content of atomically dispersed Fe (4.6 wt.%) in Ce/Fe-NCNW is achieved by the bonding of Fe atoms to O in the lattice during heat treatment and the effective suppression of agglomeration of isolated Fe atoms, made possible by ceria’s spatial confinement and strong trapping in this process. In addition, the synergistic effect of Fe and Ce further improves O_2_ adsorption and reduction kinetics, leading to enhanced ORR activity.

**Figure 8:**
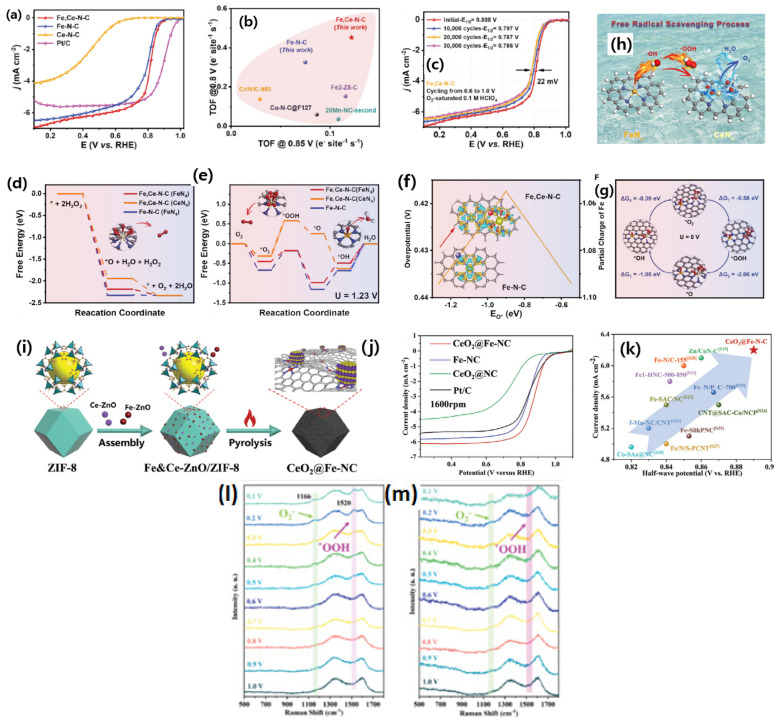
(**a**) LSV curves of FeCe-N-C catalysts in O_2_ saturated 0.1 M HClO_4_. (**b**) Current density and TOF (measured at 0.8 V vs. RHE) of FeCe-N-C, and other recently reported M-N-C catalysts. (**c**) LSV curves of various FeCe-N-C catalysts before and after accelerated degradation test (ADT) for 30,000 with theoretical calculations of the superior performance of FeCe-N-C catalysts. (**d**) Optimized atomic structures for the main process of Fenton reactions with different sites. (**e**) Gibbs free energy profiles for ORR at U = 1.23 V. (**f**) Relationship between the ORR overpotential, the binding energy of O*, and the partial charges of Fe, calculated by Bader charges. The cyan and yellow sections illustrate electron consumption and cumulation, respectively. (**g**) The mechanism schemes on FeN4 sites of FeCe-N-C at U = 0 V. (**h**) Radical scavenging mechanism Ref. [104]. (**i**) Schematic illustration for construction of CeO_2_@Fe–NC based on ZIF-8. (**j**) ORR curves at 1600 rpm in 0.1 m KOH. (**k**) Activity comparison of CeO_2_@Fe-NC with many representative catalysts and Raman characterizations of the CeO_2_@Fe-NC and Fe-NC catalysts. (**l**,**m**) In situ Raman spectroscopy of CeO_2_@Fe-NC in O_2_-saturated 0.1 m KOH and in situ Raman characterization of the Fe-NC and FeCe-NC catalyst in O_2_-saturated 0.1 M KOH Ref. [105].

Though Fe-N-C-based catalysts are sought for as the best alternatives to the traditional Pt/C catalysts, they suffer from poor stability due to surface carbon oxidation, demetallation, protonation of the N-groups, and anion adsorption. One of the greatest limitations of Fe-N-C-based catalysts is the formation of reaction oxygen species (ROS) such as ^●^OH, which irreversibly destroys the carbon support and membrane. Carbon support corrosion leads to the demetallation or loss of FeN_x_ active sites. To mitigate the effect of ROS, Chu et al. [104] designed atomically dispersed Fe and Ce atoms on the microporous carbon derived from the MOFs. While synthesizing the Fe/Ce through ZIF-8 strategy, the presence of Ce^3+^ ions could dynamically alter the growth of ZIF-8 and significantly alters (decreases) the particle size. This feature may be linked to the comparatively reduced growth rate of ZIF-8 in the presence of Ce, resulting in a smaller particle size and enhanced encapsulation of Fe ions within the framework. Consequently, in addition to the potential radical scavenging effect, the introduction of Ce enhances catalytic activity through effective Fe incorporation (augmenting active site density) and ZIF-8 particle size reduction (enhancing Fe utilization). DFT studies suggest that the reaction of H_2_O_2_ decomposition to O_2_ and H_2_O are similar on both FeCe-N-C and Fe-N-C catalysts. However, a significant difference is observed in the potential energy diagram of the FeCe-N-C catalyst, in which Ce sites catalyzing the *O to O_2_ step are thermodynamically faster, which is beneficial to reduce ROS conversion into H_2_O. Furthermore, the Gibbs free energy profiles suggests that the FeCe-N-C catalyst showed the lowest energy barrier for facilitating the adsorbed *OH species from the Fe site. Exploration of the electronic density distribution through Badar charge analysis reveals lower adsorption energy for oxygen species on the FeCe-N-C catalyst due to electron delocalization between Fe and Ce. The independent active sites of CeN_x_ are highly active towards catalyzing OOH* (to yield O_2_*) and O* (to yield OH*). This DFT analysis clearly reveals the participation of Ce on the radical scavenging of ROS, thereby protecting the Fe-Nx active sites. The experimentally synthesized catalysts showed that FeCe-N-C catalysts exhibited enhanced ORR activity in the order of Fe,Ce-N-C > Fe-N-C >> Ce-N-C with the half-wave potential of 0.808, 0.791, and 0.454 V vs. RHE, respectively, in 0.1 M HClO_4_. Experimental and direct evidence of the FeCe-N-C catalyst could be deduced from the RRDE experiments and estimation of H_2_O_2_, which is found to be <1% in all the potential range, suggesting that the FeCe-N-C catalyst is a great choice for the ORR. In addition, the effect of Ce radical scavenging can also be translated from the stability test, in which the FeCe-N-C catalyst presented excellent stability with just a loss of 22 mV after 30,000 potential cycles, better than the Fe-N-C catalyst and Pt/C catalyst, suggesting that Ce radical scavenging helps to enhance the stability of the catalysts. The experiment and theoretical analyses indicate that Ce single sites promote catalysis, scavenging ROS and promoting the 4e ORR process by decomposing H_2_O_2_ (Figure 8a–h).

In another study, there is a similar approach to improving electrocatalytic activity and stability by introducing CeO_2_ as an antioxidant agent, presented by Luo et al. [105]. An interesting in situ Raman spectroscopy analysis revealed the gradual appearance of Raman shift at 1166 and 1520 cm^−1^, corresponding to the O_2_¯ and *OOH intermediates as the potential rises from 1.0 to 0.1 V for CeO_2_@Fe-NC, whereas these Raman shift peaks are barely visible in the case of Fe-NC catalysts, indicating a definite role of Ce in enhancing ORR activity. Theoretical simulations indicate a strong electronic interaction between Fe-N_x_ moieties and CeO_2_. The Gibbs free energy for the RDS of *OH was found to be 0.39 eV for the CeO_2_@Fe-NC catalyst, whereas it was 1.01 eV for the Fe-NC catalyst, indicating that the presence of CeO_2_ enhances the ORR activity significantly. Similar observations were drawn from the experimental studies, in which the CeO_2_@Fe-NC catalyst showed a half-wave potential of 0.89 V vs. RHE. The number of electrons transferred per O_2_ molecules was found to be four, suggesting that CeO_2_@Fe-NC ORR proceeds by a direct four-electron pathway. The H_2_O_2_ UV-Vis absorption spectra shows that H_2_O_2_ begins to decompose at 80 s for the CeO_2_@Fe-NC catalyst, whereas it is 225 sec in case of the Fe-NC catalyst, indicating that CeO_2_ played a crucial role in eliminating H_2_O_2_ during ORR (Figure 8i–m). In another study [106], it was found that Ce incorporation into CL-Fe/(Fe,Ce)_x_O_y_-NC enhances the concentration of active sites, including pyridinic nitrogen, graphitic nitrogen, and metal-chelated nitrogen. The CL-Fe/(Fe,Ce)_x_O_y_-NC shows both a micro- and mesoporous nature, with a specific surface area of 891 m^2^ g^−1^. XPS analysis reveals that presence of Ce^3+^ (5s^2^5p^6^4f^1^) can interact positively with the intermediate p electrons of the reaction intermediates, promoting charge transfer and proton coupling, whereas the Ce in the form of Ce^4+^ with the electronic configuration of Ce^4+^(5s^2^5p^6^) is completely occupied, and the internal electronic structure is closed, resulting in poor catalytic activity. Therefore, the electrocatalyst with high Ce^3+^ results in enhanced ORR activity. The resulting CL-Fe/(Fe,Ce)_x_O_y_-NC exhibits a *E*_1/2_ of 0.861V vs. RHE and acceptable stability.

In a unique study, Zhang et al. [107] introduced cerium oxide cyanamide (Ce_2_O_2_CN_2_) into the Fe-N-C active sites due to the presence of unique (N=C=N)^2−^, which can enhance the conduction of the carbon support through a conjugation effect. Since the (N=C=N)^2−^ group has a lower electronegativity than metal oxides such as CeO_2_, it is possible to alter the distribution of electronic clouds, which in turn affects the material’s charge transfer capability. Therefore, the cerium oxide cyanamide can act as promoter to improve the catalytic activity and stability of M–N–C catalysts. Based on these assumptions, a heterostructure of Fe–N–C@Ce_2_O_2_CN_2_ has been synthesized. As expected, the Fe–N–C@Ce_2_O_2_CN_2_ catalyst showed remarkable electrocatalytic performance and methanol tolerance for the ORR, surpassing that of Fe-N-C and commercial Pt/C, due to the surface oxygen density modulation ability of the Ce^4+^/Ce^3+^ redox pair and the high density of the Fe-N_x_ active sites. The Fe–N–C@Ce_2_O_2_CN_2_ catalyst outperforms the Pt/C catalyst in terms of ORR activity, with a half-wave potential (E_1/2_) of 0.89 V vs. RHE in a 0.1 M KOH solution. In addition, in Zn–air batteries, the catalyst shows a much higher peak power density (119.35 mW cm^2^) than commercial Pt/C (80 mW cm^2^).

In brief, Ce is shown to be in charge of Fe’s electronic structure regulation as well as for spatially confining and stabilizing Fe atoms. Because 4f^1^ (Ce^3+^) localized electron transfer to d-orbitals of the Fe active site is due to their difference in electronegativity values in electrons, transfer from Ce → Fe further enhances the interaction of O_2_ on Fe active sites. Ce 4f^1^ of (Ce^3+^) has great affinity for interacting with transition metal atoms such as Fe. By lowering the band gap and improving conduction band dispersion, this hybridization of 4f-3d orbitals promotes effective ORR. Therefore, it is important to have a high ratio of Ce^3+^ in the catalyst. It was observed that hybridizing d-f orbitals reduces the d-band center of the Fe atom, so facilitating easy transfer of electrons to the adsorbed OH intermediates and accelerates the rate-determining step and hence the ORR. Consequently, it can be deduced that in Fe-Ce dual-atom-based catalysts, Fe is the main active center and rare earth Ce 4f electrons help to improve the charge transfer and hence reduce the d-band center and thus enhance the ORR kinetics.

## 5. Co/CeO_2_/C Catalysts for ORR

Due to their lower radical oxygen species production, Co-N-C catalysts have gained tremendous attention as an alternative to of Fe-N-C. Since Co-N-C electrocatalysts are inactive for Fenton-like reactions and stable under harsh conditions, they have been pursued as ORR catalysts. Due to modified catalyst electron structure, O_2_ storage capacity, and shift in the d-band center, combining Co and Ce could improve ORR activity. Furthermore, the O_2_ buffering (store/release mechanism of oxygen) capacity of CeO_2_ can further be tuned by choosing alternative Ce precursors to the traditional Ce nitrates.

**Figure 9:**
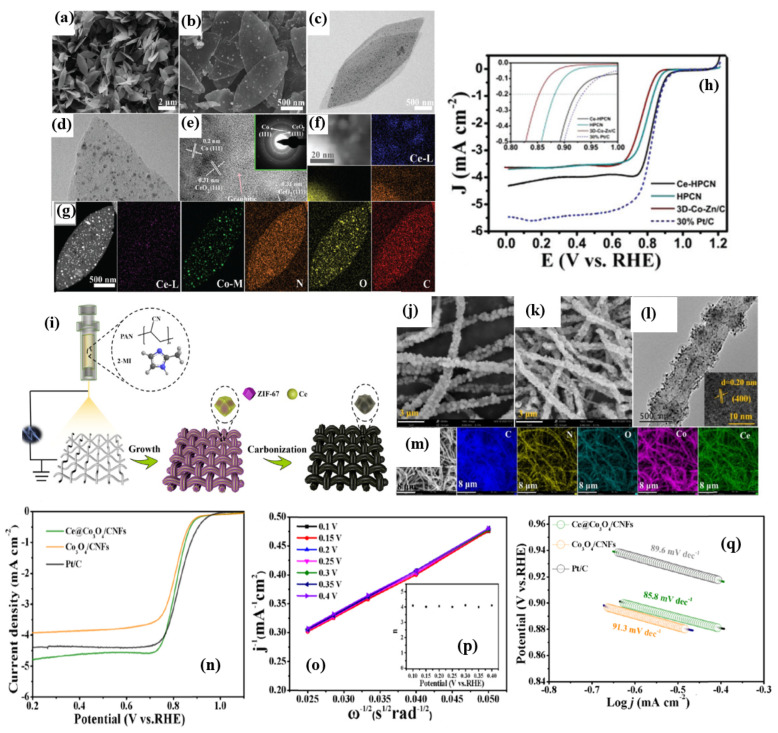
(**a**,**b**) SEM and (**c**–**g**) TEM images and elemental mapping of different elements of Ce–HPCN. (**h**) ORR curves of Ce–HPCN catalysts Inset of (**e**) shows the SAED picture of Co and CeO_2_. (**f**) ORR LSV curves of 3D-Co–Zn/C, HPCNs, and Ce–HPCNs catalysts @ 1600 rpm (Reproduced with permissions from Ref. [108]). (**i**) Schematic representation of synthesis of Ce@Co_3_O_4_/CNFs. (**j**–**m**) SEM and TEM images and elemental mapping of Ce@Co_3_O_4_/CNFs catalysts. (**n**) LSV curves. (**o**) K-L plots. (**p**) Number of electrons. (**q**) Tafel plots of Ce@Co_3_O_4_/CNFs catalyst (Reproduced with permissions from Ref. [116]).

Xia et al. [108] found that by adding a Ce_2_(OH)_4_SO_4_.2H_2_O precursor during the nucleation process of ZIF, 2D-hexagonal-leaf-like ZIF lamellae (ZIF-L) can be obtained which could generate more oxygen vacancies than by using traditional Ce precursors such as Ce nitrates (Figure 9a–g). The leaf-like ZIF-L structures are formed by careful control of the 2-MIM and Co^3+^ and Zn^2+^ ions. In general, the amine N-groups from the 2-MIM interact with the H atoms of H_2_O, forming H-bonds and bridging 2MIM-N-H bonds, making it further extended with other molecules of N-2MIM through sodalite layers, leading to the formation of leaflike structures. Based on this assumption, the leaf-like 2D ZIF-like structures have been observed to have a unique, smooth surface. Although the surfaces of the leaf-like lamellae become rough and numerous nanoparticles (white dots) are embedded in the surfaces of the Ce-HPCNs, the pristine leaf-like morphology of the Ce-HPCN precursor is preserved in the resulting Ce-HPCNs, as anticipated. The HR-TEM analysis shows clear leaf-like structures, with Co and CeO_2_ nanoparticles in close contact with each other. The RDE studies further showed that the ORR onset potential of 0.923 V and a half-wave potential of 0.831 V for Ce-HPCN are higher than the Pt/C catalysts, due to ther unique leaf-like morphology, highly 2D open pore structure, higher content of pyridinic-N and graphitic-N sites, and the synergistic effect of Co-N_x_, along with the oxygen buffering properties of CeO_2_ (Figure 9h). The O_2_ buffering capacity of Ce is further determined from the O_2_ temperature-programmed desorption (O_2_-TPD) spectra, which clearly show a high-intensity O_2_ desorption peak, indicating the enhanced adsorption energy of O_2_ for the sample of Ce–HPCNs [109]. All of these findings point to the possibility that doped CeO_2_ could significantly improve O_2_ adsorption capacity and the interaction between Co-N_x_ active sites, leading to outstanding ORR performance.

Mixed metal oxides are known examples of synergistic catalysts, in which two types of dissimilar metal oxides are known to improve electrochemical properties. For instance, a heterostructure of the CO_3_O_4_-MnO_2_/C catalyst delivered enhanced ORR activity, owing to a direct four-electron reduction of O_2_ as a result of covalent coupling between Co_3_O_4_-MnO_2_ [110]_._ Similarly, the Pd@PdO-CO_3_O_4_ heterostructure catalysts delivered high ORR/OER activity [111]. In the same way, coupling CO_3_O_4_ with a rare earth CeO_2_ could be the best choice, owing to their abundant oxygen vacancies and oxygen storage capacity, along with reversible surface ion exchange capacity, leading to enhanced ORR activity. Li et al. [112] synthesized Co_3_O_4_@Z67-NT@CeO_2_ by a sequential synthesis process in which CeO_2_ nanoparticles were introduced onto the surface of Co_3_O_4_@Z67-NT by a hydrothermal synthesis method. The XRD analysis revealed characteristic diffraction peaks of both Co_3_O_4_ and CeO_2_, indicating the crystalline nature of bi-metal oxides in the catalysts. The XPS analysis showed the presence of high pyridinic-N, which is responsible for enhancing electronic conductivity, and onset potential for ORR, whereas graphitic-N content is known to enhance the limiting current density [113,114]. The XPS analysis of Co_3_O_4_@Z67-NT@CeO_2_ shows a high proportion of Ce^2+^, which are responsible for high ORR activity, along with Ce^3+^ ions. Furthermore, the electrostatic interaction between Co and Ce is ascertained from the positive shift in the binding energy of Ce^3+^. The relative atomic composition of Ce^3+^ and Ce^4+^ hint at the reason behind the ORR activity of Co_3_O_4_@Z67-NT@CeO_2_ catalysts. It was found that the relative abundance of Ce^3+^ is about 36.91%, indicating that the ORR activity of the Co_3_O_4_@Z67-NT@CeO_2_ catalyst is due to the role of charge compensation by Ce^3+^, which will generate oxygen vacancies to promote the oxygen adsorption on the catalyst surface [115]. Furthermore, the continuous availability of a higher valance state of Co is possible due to the lattice oxygen provided by the CeO_2_, keeping the catalytic cycle happening continuously. The ORR half-wave potential of Co_3_O_4_@Z67-NT@CeO_2_ is found to be higher than Co_3_O_4_@Z67-NT by 20 mV, being 0.88 V vs. RHE in 0.1 M KOH electrolyte, indicating that CeO_2_ guarantees enhanced activity.

In another study that examined the effect of rare earth metal oxide, CeO_2_ was combined with Co_3_O_4_, gaining the advantage of CeO_2_ as a redox couple and oxygen buffering agent. In a three-step strategy, Ce is introduced into the carbon nanofibers loaded with Co_3_O_4_ (Ce@Co_3_O_4_/CNFs) using an electrospinning (Figure 9i) method [116]. The XRD analysis reveals almost no visible CeO_2_ peaks, indicating that either Ce is doped into the Co_3_O_4_ or dispersed on the catalyst due to low Ce content. This is further confirmed when Ce atoms have no effect on the polyhedral morphologies of MOF–fiber composites. TEM images show the highly dispersed, tiny Co_3_O_4_ nanoparticles evenly dispersed on the nanofiber surface, ensuring abundant exposed active sites for ORR (Figure 9j–m). The XPS analysis revealed that Co^3+^/(Co^3+^ + Co^2+^) molar ratio increases in Ce@Co_3_O_4_/CNFs, indicating the possibility of Ce atoms being in close contact with Co_3_O_4_ or inserted into the crystal structure of Co_3_O_4_. Furthermore, the significantly increased Co^3+^ species infer that the electronic structure of Co in Co_3_O_4_ can be easily fine-tuned by introducing the Ce species, owing to their redox coupling reaction between Co^3+^/Co^2+^ and Ce^3+^/Ce^4+^. Moreover, the presence of a high proportion of Ce^3+^ is found to be beneficial for electrocatalytic activity [117]. The ORR curves clearly establish the synergistic effect of Co_3_O_4_ and Ce on the ORR activity as the Ce@Co_3_O_4_/CNF catalyst shows almost similar activity as that of the commercial Pt/C catalyst (Figure 9n–q). The Ce@Co_3_O_4_/CNFs ZAB exhibits remarkable electrocatalytic activity, which can be attributed to the combination of Co_3_O_4_ and CeO_2_ as well as the high conductivity of CNFs.

**Figure 10:**
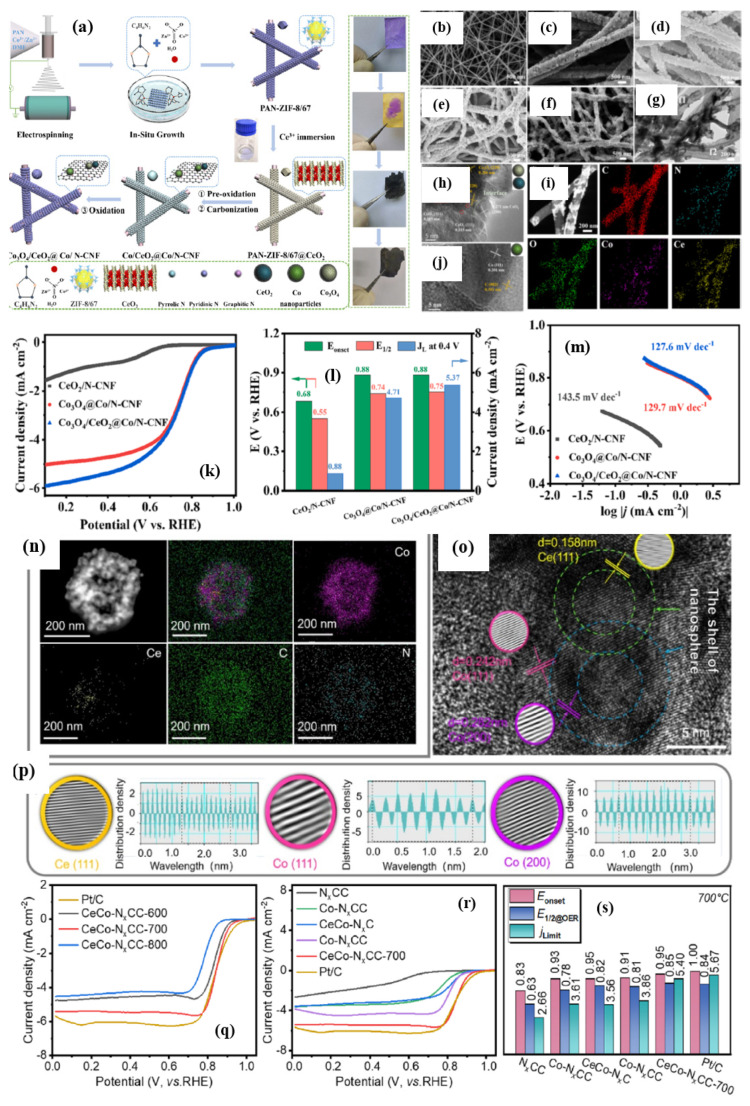
(**a**) Schematic representation of Co_3_O_4_/CeO_2_@Co/N-CNF catalyst synthesis. (**b**–**j**) SEM and TEM images of Co_3_O_4_/CeO_2_@Co/N-CNF catalyst and the colored elemental mapping images. (**k**) LSV (**l**) histogram representing Eonset, E1/2, and JL. (**m**) Tafel plots of Co_3_O_4_/CeO_2_@Co/N-CNF catalysts (Reproduced with permissions from Ref. [121]). (**n**,**o**) STEM elemental mapping and HRTEM images of the CeCo-NxCC-700 catalysts. (**p**) Digital micrographs of Ce(111), Co(111), and Co(200) on image (**o**) of the CeCo-NxCC-70 catalyst. (**q**,**r**) ORR LSV curves recorded in 0.1 M KOH electrolyte of CeCo-N_x_CC catalysts. (**s**) Histogram representing the onset, half-wave, and JL of CeCo-N_x_CC catalysts (Reproduced with permissions from Ref. [130]).

Metal oxides such as Co_3_O_4_-based spinel oxides, which are a kind of *p*-type semiconductor, have been explored as efficient ORR catalysts due to their unique crystal structure in which Co^2+^ occupies tetrahedral sites and Co^3+^ occupy octahedral sites [118,119]. CeO_2_, a rare earth based on Ce, is an *n*-type semiconductor that is an interesting metal oxide due to its high ionic/electronic conductivity and oxygen storage capacity and can influence the oxygen reduction catalysis [120]. Because of the strong electronic coupling effect, it is believed that hybridizing *p*-type Co_3_O_4_ and *n*-type CeO_2_ will increase the activity of oxygen catalysis. By combining *n* and *p*-type materials, engineers can design and fine-tune an electrical structure that is advantageous for optimizing surface properties for enhanced ORR. Guo et al. [121] synthesized a Co_3_O_4_/CeO_2_ heterostructure in situ embedded in Co/N-doped carbon nanofibers through a facile electrospinning process (Figure 10a–j). The bimetallic ZIF grows in a tiled pattern due to the attractive force of metal ions on the surface of the nanofibers, which facilitates the cross-linking with 2-MIM. The following in situ secondary growth method is then used to synthesize regular ZIF nanoparticles. The subsequent hydrolysis of Ce^3+^ in the presence of oxygen from the surrounding environment yields CeO_2_ product, which is then loaded onto the surface of the fiber and appears yellow. After that, annealing in an Ar environment yields a composite of Co_3_O_4_/CeO_2_@Co/N-CNF and an air environment yields a composite of the same composition. A distinct heterointerface structure was observed in the synthesized Co_3_O_4_/CeO_2_@Co/N-CNF.

At the interface, there was a distinct boundary between the two phases, with distinct groups of lattice fringes with interplanar spacings of 0.286 and 0.271 nm, representing the (220) and (200) planes of Co_3_O_4_ and CeO_2_, respectively. The heterointerface between Co_3_O_4_ and CeO_2_ is a clear indication of the strong interfacial electronic interaction between them. The XPS analysis reveals the presence of Co in the form of metallic, Co^2+^ and Co^3+^ and Ce in the form of Ce^3+^/Ce^4+^ oxidation states. The O_vac_ calculated from the O1s spectra; it was found that the O_vac_ ratio is found to be higher for the Co_3_O_4_/CeO_2_@Co/N-CNF catalyst compared to Co_3_O_4_@Co/*N*-CNF, indicating the significance of CeO_2_ in creating the O_vac_ in the catalyst. The unsaturated coordination environment is conducive to electron delocalization, and it has been generally reported that oxygen vacancies can accelerate the charge transfer rate by promoting more carriers to pass through. Also, oxygen vacancies can control the adsorption energy of the intermediate and enhance oxygen adsorption, which can lead to more efficient ORR kinetics.

According to the DFT studies by Li et al. [122], the oxygen adsorption on the defected oxygen vacancy metal oxides is ascribed to less filling of the antibonding states. In other words, the presence of oxygen defects moves the *d*-band center towards the Fermi level, indicating enhanced bond strength and adsorption of oxygen to the metal oxide surface. Therefore, the more oxygen vacancies, the better the electrocatalytic properties of the catalysts. The Co_3_O_4_/CeO_2_@Co/N-CNF catalyst was found to have more oxygen vacancies; therefore, it can deliver better ORR kinetics. Accordingly, the Co_3_O_4_/CeO_2_@Co/N-CNF catalyst presented a higher onset and half-wave potential of 0.88 V and 0.75 V, respectively, higher than the counter catalysts such as the Co_3_O_4_@Co/N-CNF and CeO_2_/N-CNF catalysts (Figure 10k–m).

Metal–organic framework-derived catalysts benefit from structural and morphological aspects such as the derived catalysts generally exhibiting large specific surface area and excellent porosity [123,124]. However, one of the persistent problems with MOF-derived catalysts is structural collapse and their susceptibility to agglomeration during the pyrolysis process [125]. Polymer surface functionalization of the ZIFs stands out to be one of the best strategies for mitigating structural collapse and agglomeration [126,127]. Among several polymers, conducting polymers such as Polyacrylinitrile and polypyrrole (PPy) are very beneficial for enhancing the ORR activity [128,129]. Tang et al. [130] described an efficient strategy in which Co-ZIFs are surface-functionalized with PPy precursor to develop hierarchical CeCo-N_x_-CC catalysts for ORR (Figure 10n–p). The synthesis process involves three steps, starting with ZIFs synthesis from Co^2+^ and 2-MIM, after which Ce^3+^ ions are doped into the Ce-ZIFs by a solution incorporation strategy; finally, the surface functionalization of Co-Ce-ZIFs is conducted by polymerization of PPy. Further heat treatment of the Co-Ce-ZIF@PPy leads to the target catalyst CeCo-N_x_CC. In this work, interestingly, the role of Ce^3+^ as structural adjusting agent is established. The authors claim that the ZIF-67 particles appeared to be spherical initially, but thereafter, with the addition of Ce, promoted the formation of a dodecahedral structure. The TEM results reveal that the CeCo-N_x_CC-700 catalyst displays a dodecahedral hollow frame morphology; as expected, the dodecahedral structure remains un-collapsed, indicating the importance of PPy functionalization for structural tuning. The XRD and XPS analysis reveal the possibility of metal–N_x_ species in the catalyst as potential ORR catalytic centers. During RDE studies, the CeCo-N_x_CC-700 catalyst showed excellent ORR activity, with an onset potential of 0.95 V and a half-wave potential of 0.85 V vs. RHE, much higher than the catalyst prepared without PPy coating and Ce incorporation. This is further confirmed by the high ECSA value of the CeCo-N_x_CC-700 catalyst, which is 8.32 mF cm^−2^ higher than the catalyst without PPy coating and Ce incorporation, indicating that both PPy and Ce incorporation helped in enhancing the ORR active sites. In addition to the enhanced ORR activity, the CeCo-N_x_CC-700 catalyst presented excellent stability with a loss of just 10 mV in half-wave potentials when compared to the 20 mV loss in half-wave potential for Pt/C, for 8000 cycles, suggesting that N-doped carbon derived from the PPy helps to mitigate the nanoparticle agglomerations during a potential cycling test (Figure 10q–s).

**Figure 11:**
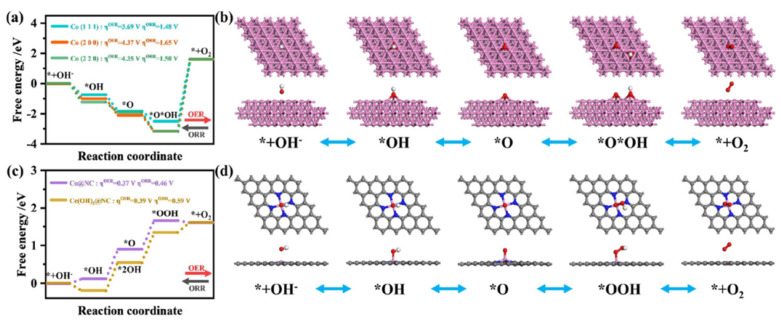

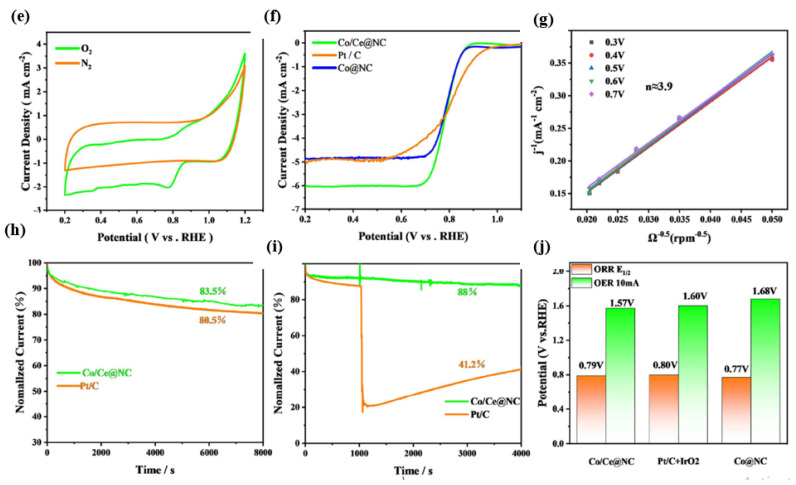
Free energy images of (**a**) Co NPs and (**c**) Co/Ce@NC and the reaction intermediates of (**b**) Co (111) and (**d**) Co@NC. (**e**) CV. (**f**) LSV of (0.1 M KOH electrolyte). (**g**) K-L plots. (**h**) *i-t* curves. (**i**) Methanol sensitivity. (**j**) Histograms showing the relationship between ORR E_1/2_ and OER potentials at 10 mA cm^−2^ (Reproduced with permissions from Ref. [131]).

In another study, Ce doping is found to be beneficial, not only for increasing the ORR activity but also in enhancing the stability of the transition metal catalysts, such as the Co@NC catalyst. A DFT study by Liu et al. [131] demonstrated that for the Co@NC catalyst, excessive energy is required for the adsorption of O_2_, which also requires a higher amount of heat adsorption for three elementary reactions (*O*OH → *O → *OH → * + OH^−^), indicating that Co alone is not ideal for efficient ORR kinetics. When Ce atoms are introduced Co/Ce@NC, it was found that the kinetics enhances due to the involvement of Ce 4f electrons. It was found that with the introduction of Ce, optimal adsorption of the O_2_ intermediates and quick description of the products have been observed. In addition, the addition of Ce was found to have a stability effect on the irreversible aggregation of atomic cobalt sites into Co nanoparticles, with the help of assessing the binding energy (Δ*E*_b_) between Co/Ce single atoms and carbon-based defect vacancies. In general, higher Δ*E*_b_ values represent the stable atomic positioning of the metallic active sites in the carbon system. The obtained Δ*E*_b_ values are 7.66 and 9.25 eV for Co@NC and Ce@NC catalysts, respectively. This clearly indicates that Ce atoms have high stability. In addition, cohesive energiy (Δ*E*_c_) is the energy required to form metal-to-metal bonds; for both Co and Ce, this is around 5.4 eV [132,133]. For Co atoms, the Δ*E*_b_ is lower than the Ce; the Co atoms are prone to undergo agglomeration, whereas for the high Δ*E*_b_ of Ce with stronger binding energy, Ce-doping into the catalysts increases the distance between the Co atoms and hence acts as a dispersing agent and stabilizing agent, effectively mimicking the Co aggregation and hence increasing the overall stability of the electrocatalyst. Based on the DFT assumption, the authors synthesized the Co/Ce@NC catalyst and investigated the effect of ORR and Ce’s role as an ORR-enhancing element (Figure 11a–d). The SEM and TEM images provide evidence of the presence of dodecahedron structures of the Co/Ce@NC catalyst along with the CNTs grown on the surface. It was also seen that some of the Co nanoparticles are encapsulated by carbon in the form of CNTs. XRD results show Co present in the metallic state. XPS results show the presence of Ce in the form of Ce^3+^/Ce^4+^. Peaks around 896 and 915 eV in the high-resolution Ce 3d XPS spectrum of Co/Ce@NC are ascribed to the Ce^3+^ and Ce^4+^ species. Interconversion between Ce^4+^ and Ce^3+^ leads to enhanced charge redistribution on Co, which results in enhanced ORR kinetics. The Co/Ce@NC catalyst in RDE studies delivered a half-wave potential of 0.80 V, higher than the Co@NC at 0.77 V, along with the lowest Tafel slopes of 64 mV dec^−1^. Furthermore, the Co/Ce@NC catalyst generated the lowest % of peroxide at 3.17%, with a n = 3.94 indicating excellent ORR activity (Figure 11e–j).

Another family of electrocatalysts that is abundant in nature and has great catalytic activity are transition metal oxides, phosphides, and sulfides, which can include elements like nickel, iron, and cobalt. Co_9_S_8_ has recently drawn a lot of interest in the energy field due to its catalytic activity towards OER. Its ORR catalysis still requires improvement though, because of the disadvantage of poor conductivity, low surface area, and its susceptibility to aggregation during the electrochemical reaction [134,135]. Because of its rich oxygen vacancies and multivalent electron transfer, CeO_2_ may effectively encourage electrocatalytic reactions [136,137]. Sun et al. [138] presented a strategy to synthesize a heterostructured Co_9_S_8_/CeO_2_/Co-NC catalyst as an excellent strategy to improve the catalytic performance of Co_9_S_8_. The resulting Co_9_S_8_/CeO_2_/Co-NC electrocatalyst demonstrated a positive half-wave potential for ORR (0.875 V vs. RHE), nearly affecting ORR activity with equal effect to the commercial Pt/C catalyst, thanks to the special heterostructure of Co_9_S_8_/CeO_2_ and its synergistic effects with Co. This study emphasizes the effect of Ce as a structural directing agent. The SEM morphology of Co-ZIF shows a spherical 3D structure, whereas Co/Ce-ZIF shows a 2D sheet morphology, suggesting the phase transition ability of Ce atoms in the ZIF structures. After pyrolysis, the SEM images show numerous nanotubes grown on the surface of 2D Co/Ce-ZIF. After a sulfidation process, a 2D Co_9_S_8_/CeO_2_/Co-NC electrocatalyst, i.e., a “senbei”-like nanosheet structure was obtained. The TEM measurements of the 2D Co_9_S_8_/CeO_2_/Co-NC catalyst show well-dispersed metallic Co and Co_9_S_8_ nanoparticles in close conjunction with CeO_2_, forming a compact heterostructured catalyst. XPS analysis clearly showed that CeO_2_ affects the charge distribution on the surface of Co_9_S_8_ due to the observable shift in the XPS analysis by 0.17 eV for Co_9_S_8_/CeO_2_/Co-NC when compared to Co_9_S_8_/Co-NC. The ratio of Ce^3+^/Ce^4+^ is an important indicator of the oxygen buffering capacity of CeO_2_, due to its ability to interexchange between the oxidation states. The Ce^3+^/Ce^4+^ ratio for Co_9_S_8_/CeO_2_/Co-NC is ∼0.27, that of CeO_2_/Co-NC is∼0.25. Electron interaction in the heterostructure may enhance the conversion from Ce^4+^ to Ce^3+^, as indicated by the rise in the Ce^3+^/Ce^4+^ ratio [139]. Unsaturated chemical bonds are formed inside CeO_2_ as a result of Ce^3+^, creating a charge imbalance. The formation of numerous oxygen vacancies as a result of charge compensation improves ORR performance and encourages oxygen adsorption on the catalyst surface [140,141]. The RDE studies show that the Co_9_S_8_/CeO_2_/Co-NC catalyst exhibited a half-wave potential of 0.875 V, much higher than the CeO_2_/Co-NC (0.83 V) and Co_9_S_8_/Co-NC catalysts (0.85 V). The high half-wave potential values of the Co_9_S_8_/CeO_2_/Co-NC catalyst indicates the heterostructured morphology and presence of CeO_2_ and Co_9_S_8_ and that the synergistic effect between the three components of metallic Co, CeO_2_, and Co_9_S_8_ plays a significant role in enhancing ORR activity. The enhanced ORR activity is further ascertained from the lowest Tafel slope of 56 mV dec^−1^ and the average number of electrons of 4.0 and 3.8 electrons from K-L plots and RRDE measurements, respectively. The % of peroxide yield of Co_9_S_8_/CeO_2_/Co-NC catalyst is 8.3%, which is the lowest of the CeO_2_/Ce-NC (23%) and Co_9_S_8_/Co-NC (9.8%) catalysts. Furthermore, the Co_9_S_8_/CeO_2_/Co-NC catalyst also showed admirable stability under potential cycling conditions, with a loss of just 10 mV in the half-wave potential, whereas Pt/C lost about 50 mV. In chronoamperometric analysis at 0.8 V for 30,000 s, the Co_9_S_8_/CeO_2_/Co-NC catalyst lost just 9% of the relative current, whereas Pt/C lost about 25% of the relative current, indicating that the Co_9_S_8_/CeO_2_/Co-NC catalyst possess higher ORR activity.

To conclude briefly, it was found that the relative abundance of Ce^3+^ is the prime factor responsible for enhancing the ORR activity of Co-based catalysts due to its role in charge compensation which generates oxygen vacancies to promote the oxygen adsorption on the Co-N-C catalyst surface. Furthermore, the continuous availability of a higher valance state of Co is possible due to the lattice oxygen provided by Ce/CeO_2_, keeping the catalytic cycle occurring continuously. From the significantly increased Co^3+^ species observed in Co-based catalysts, it can be inferred that the electronic structure of Co in Co_3_O_4_ can be easily fine-tuned by introducing the Ce species, owing to their redox coupling reaction between Co^3+^/Co^2+^ and Ce^3+^/Ce^4+^. Because of the strong electronic coupling effect, it is believed that hybridizing *p-type* Co_3_O_4_ and *n-type* CeO_2_ will increase the activity of oxygen catalysis. By combining *n-type* and *p-type* materials, engineers can design and fine-tune an electrical structure that is advantageous for optimizing surface properties for enhanced ORR, which is experimentally observed. The presence of oxygen defects in CeO_2_ moves the *d*-band center towards the Fermi level, indicating an enhanced bond strength and adsorption of oxygen to the metal oxide surface, thereby promoting the Co-based catalysts to exhibit improved ORR performance.

## 6. Atomically Dispersed Ce-Based and CeO_2_ Catalysts for ORR

The role of Ce in enhancing electrocatalytic activity is clearly seen in the case of the Pt/CeO_x_, Fe/CeO_2_/C, and Co/CeO_2_/C catalysts for ORR. However, several studies have shown that pristine CeO_2_ in combination with N-doped carbon itself could possess admirable ORR activity. This is because, due to the abundant oxygen vacancies and quick transition between Ce^3+^ and Ce^4+^, Ce itself can act as ORR active site. In addition, when compared to Ce in the form of CeO_2_ nanoparticles, the atomically dispersed Ce catalysts, commonly known as single-atom catalysts (SACs), have gained tremendous popularity in recent years due to their homogeneous dispersion, well-defined active sites, efficient metal atom utilization and unique co-ordination environment in the catalysts. This section describes the recent trends in the intrinsic catalytic activity of CeO_2_ in combination with N-doped carbon and atomically dispersed Ce-N_4-_C catalyst ORR activity.

Yu et al. [142] demonstrated the use of a CeO_2_@ZIF-8-derived CeO_2_@N-doped carbon as an ORR catalyst. The synthesis process included the CeO_2_ encapsulation of ZIF-8 in the presence of surfactant PVP, leading to the trapping of the CeO_2_ nanoparticles inside the ZIF-8 structures. The pyrolysis temperature was found to have a direct influence on the ORR activity of CeO_2_@N-doped carbon as an ORR catalyst. The RDE studies clearly show the increase of half-wave potentials with the increase in pyrolysis temperatures, with optimum ORR activity achieved with the pyrolysis temperature of 900 °C for the catalyst CeO_2_@N-C-900, with an onset potential of 1.003 V and halfwave potential of 0.908 V vs. RHE in 0.1 M KOH electrolyte, surpassing the commercial Pt/C catalyst. In addition, the CeO_2_@N-C-900 catalyst showed an average number of electrons transferred per O_2_ molecule be 3.97, confirming the excellent ORR activity of the CeO_2_@N-C-900 catalyst. However, the CeO_2_@N-C-900 catalyst delivered a slightly lower ORR performance in acidic electrolytes. In addition to the enhanced ORR activity, the CeO_2_@N-C-900 catalyst also presented excellent durability, with almost no activity loss in 0.1 KOH and about 21 mV loss of activity in 0.1 M HClO_4_ electrolytes. In another study, Wen et al. [143] investigated the effect of CeO_2_ nanoparticle grown on N-doped carbon (CeO_2_–CN) and its ORR activity. The CeO_2_–CN nanoparticles were synthesized by a solid-state synthesis process, in which previously synthesized ZIF-8 nanoparticles are placed in a gate motor and mixed with Ce-nitrate and NaCl precursors and the resulting mixture is ground, followed by calcination and NaCl removal with H_2_O, leading to the formation of CeO_2_–CN electrocatalyst. Similarly to other studies, the authors claim that the enhanced ORR activity may be attributed to the content of Ce^3+^, evaluated from the XPS analysis, which described the role of Ce^3+^ in compensating for the oxygen vacancies, therefore indicating that the content of Ce^3+^ is directly proportional to the oxygen vacancies in the catalysts [144,145]. The CeO_2_–CN-800 catalyst showed a higher content of Ce^3+^; therefore, higher ORR activity can be expected. In RDE studies, the CeO_2_–CN-800 catalyst exhibited an onset potential of 0.90 V and a half-wave potential of 0.84 V, nearing the standard values of Pt/C which are 0.92 V and 0.85 V, respectively. In another study, in an ORR test conducted in alkaline media, cerium carbide with nitrogen-doped carbon (CeC_x_-NC) displayed a four-electron mechanism and a half-wave potential close to Pt/C [146].

When compared to Ce in the form of CeO_2_ nanoparticles, atomically dispersed Ce catalysts, commonly known as single-atom catalysts (SACs), have gained tremendous popularity in recent years due to their homogeneous dispersion, well defined active sites, efficient metal atom utilization, and unique co-ordination environment in the catalysts [147,148]. In addition, the SACs with M-N-C active sites are certainly suitable candidates as electrocatalyst due to their high electronic conductivity due to the presence of N, which exhibits excellent resistance to poisoning spices and admirable stability [149,150,151]. The best M-N-C-based catalyst so far is composed of Fe-N-C, with the potential to replace the Pt-based catalysts [152,153,154,155]. However, when Fe-N-C catalysts are formed during the high-temperature pyrolysis process, Fe co-ordinates with N (Fe-N_x_), with a variety of co-ordination numbers ranging from x = 2–6, leading to the formation of Fe-N_2_ and Fe-N_5_ in addition to Fe-N_4_. There is enough evidence regarding the electronic structure and mechanistic studies indicating that Fe-N_4_ sites are the most active and desirable ORR active sites that catalyze a direct four-electron reduction of O_2_ to H_2_O, whereas other forms of Fe-coordination sites such as Fe-N_2_ and Fe-N_5_ catalyze by a mixture of four and 2+2 O_2_ reduction pathways, which lead to the formation of H_2_O and H_2_O_2_ as the final products.

**Figure 12:**
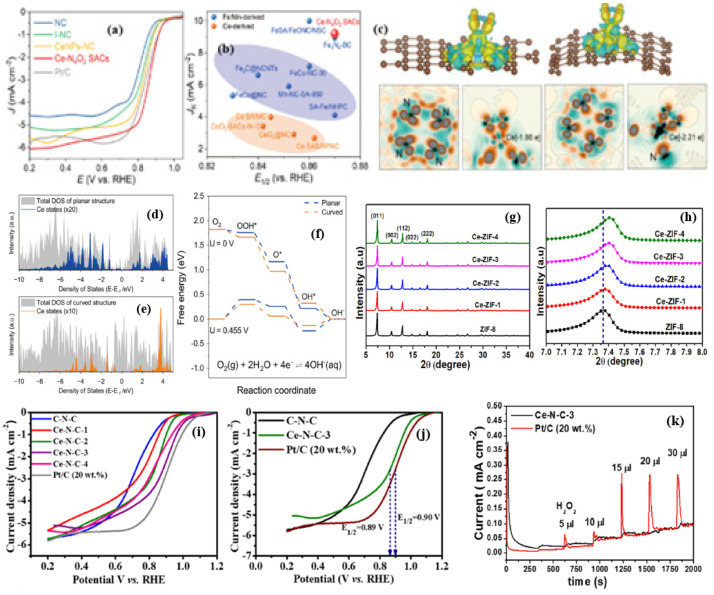
(**a**) LSV curves. (**b**) Comparison of half-wave potentials, J_k_ of the various CeN_4_O_2_ catalysts, and charge density around different N and Ce atoms of (**c**) planar and (**d**) curved CeN_4_O_2_. PDOS of (**d**) planar and (**e**) curved CeN_4_O_2_. (**f**) Free energy diagram of planar and curved CeN_4_O_2_ (Reproduced with permissions from Ref. [158]). (**g**) XRD pattens of Ce-ZIFs with different concentration of Ce. (**h**) Zoomed version of the Ce-ZIFs. (**i**) LSV curves of various Ce-N-C catalysts. (**j**) Ce-N-C, Pt/C catalysts. (**k**) Chronoamperometric curves of Pt/C and Ce-N-C-3 catalysts in response to the addition of H_2_O_2_ (Reproduced with permissions from Ref. [160]).

H_2_O_2_ is an undesirable product that reacts with the Fe^2+^ ions, leading to the formation of ^●^OH radicals that react with other catalyst components and membranes, potentially degrading them [156]. In this regard, Ce is a very interesting element which has the potential to mimic the disadvantages of Fe-N_x_-based catalysts due to the fact that Ce has a radical scavenging capacity. Ce SACs have been found to have high-spin state configurations that are potentially the active sites for ORR [156]. However, the synthesis of the higher dispersed single-atom catalysts composed of Ce is challenging due to their high surface and migration energies, leading to the formation of Ce nanoparticles during the high-temperature pyrolysis step. Furthermore, due to its high affinity towards oxygen, Ce forms Ce-oxides [157]. Therefore, controlling the synthesis conditions is a key to obtaining the Ce SACs. Liu et al. [158] proposed a unique synthesis strategy in which atomic-level dispersion of Ce achieved Ce loading of as high as 15.9% with a unique catalytic active site composed of Ce-N_4_O_2_ SACs as an excellent ORR catalyst, along with superb radical scavenging ability. A unique iodine-induced metal phase modulation strategy was developed with the help of MET-6, which is a zinc-triazolate metal–organic framework (MOF) derivative. With this unique synthesis strategy, the formation of Ce nanoparticles is greatly avoided by achieving an excellent dispersion of the Ce atoms with an atomic percentage as high as 15.9%. For the detailed synthesis process, we ask the readers to refer to the experimental section of the article. The RDE studies show that Ce-N_4_O_2_ SACs revealed a half-potential of 0.85 V, Tafel slope of 76 mV dec^−^1, and a J_k_ of 8.82 mA cm^−2^ at 0.85 V, surpassing the Pt/C catalyst. The radical scavenging capacity of Ce-N_4_O_2_ SACs catalyst is assessed with UV-vis absorption spectra (Figure 12a,b). The Ce-N_4_O_2_ SACs and Ce-NPs-NC SACs displayed lower absorbance at 417 nm, indicating that Ce-N_4_O_2_ SACs can effectively scavenge the reactive oxygen species. The ORR mechanism on the Ce-N_4_O_2_ SACs catalyst is assessed by a theoretical simulation. Two possible atomic models were considered, one of which involved placing the CeN_4_O_2_ planarly and other involving placing it at the curved location of the graphene-like carbon support. Both the planar and curved Ce-N_4_O_2_ showed dynamic stability, and both the models displayed effective electronic charge redistribution, among which curved Ce-N_4_O_2_ active sited displayed a greater charge density than the planar one. Badar charge analysis reveals 1.96 and 2.21 electrons being added from the N atoms to the Ce in planar and curved Ce-N_4_O_2_ active sites, respectively. In a free energy analysis, both planar and curved Ce-N_4_O_2_ active sites showed a downhill trend for the ORR intermediates suggesting the spontaneity of the ORR. The curved structure’s computed ORR over-potential under alkaline conditions was 0.28 eV, which was less than the planar site’s (0.40 eV) (Figure 12c–f). This displays the advantageous role that graphene curvature plays in maximizing ORR activity [159].

As previously mentioned, new research indicates that single-atom catalysts that are atomically dispersed have a different reactivity than catalysts based on nanoparticles. Instead of looking into Ce in the form of CeO_2_ nanoparticles, it is worthwhile looking into atomically dispersed Ce in N-doped carbons that are strictly confined in the carbon matrix. This is because the electrocatalytic activity can be greatly increased when the concentration of Ce species decreases from the nanoscale to the single atomic level, due to the enhanced surface free energy that aids in O_2_’s adsorption and subsequent reduction. The catalyst’s stability can be increased by fixing the cerium atoms with the aid of four coordinated -N atoms to create Ce-N_4_-C. This helps to reduce the leaching of cerium atoms. In a study, Peera et al. [160] described Ce-N_4_-C catalyst synthesis by a simple ZIF-based strategy and found that the Ce-N_4_-C catalyst possessed almost similar ORR kinetics as that of Pt/C. The successful insertion of Ce into the ZIF’s crystal lattice was confirmed from the X-ray diffraction analysis, in which the diffraction peak at 7.46° shifts to the higher 2θ angles for Ce-ZIF precursors; this is due to the ionic radius of Ce^3+^/Ce^4+^, which is larger than the Zn^2+^ ions (Figure 12g,h). The Ce-N_4_-C catalyst derived from the pyrolysis of the Ce-ZIFs presented an extraordinary BET surface area of 905 m^2/^g. The TEM images show the very small amount of Ce atoms in the atomically dispersed form. The XPS analysis shows the co-existence of Ce^4+^/Ce^3+^ in almost equal proportions, which guarantees the altering oxidation states during the ORR. In addition, the Ce^4+^ was 12% more abundant compared to Ce^3+^. While Ce^3+^ is good at scavenging ^●^OH radicals, Ce^4+^ is good at oxidizing H_2_O_2_. Therefore, the overall ORR catalytic activity is improved by the synergistic effect of the Ce^4+^/Ce^3+^ redox couple [161]. The optimized Ce-N-C-3 catalyst showed the best ORR activity, with a half-wave potential of 0.89 V vs. RHE, which is just 10 mV lower than the Pt/C catalyst, indicating that the Ce-N-C-3 catalyst could be a promising alternative to the Pt/C catalyst. Interestingly, the Ce-N-C-3 catalyst also showed the lowest H_2_O^−^ of just 4%, the lowest among several Ce-based catalysts presented in this review. The authors in this study also performed the peroxide test to confirm the ability of Ce^4+^/Ce^3+^’s redox couple scavenging capacity towards peroxide/hydroperoxide radicals. In a specialized test, H_2_O_2_ was added to the 0.1 M KOH solution under chronoamperometric conditions and current profiles were recorded for Pt/C and Ce-N-C-3 catalysts. The Pt/C catalyst showed current spiking with the addition of increasing levels of H_2_O_2_, whereas the Ce-N-C-3 catalyst showed an almost negligible rise in the currents, suggesting that the Ce-N-C-3 catalyst successfully deactivated or scavenged the H_2_O_2_/HO^•^/hydroperoxyl radical (HO_2_^•/−^) (Figure 12i–k). These findings show that Ce-based catalysts are effective at catalyzing ^●^OH scavenging reactions, which reduces carbon support corrosion, accelerates the dissolution of particles and atomic atoms, breaks down the ionomer in the catalyst layer, and damages PEM/AEM membranes in fuel cells.

**Figure 13:**
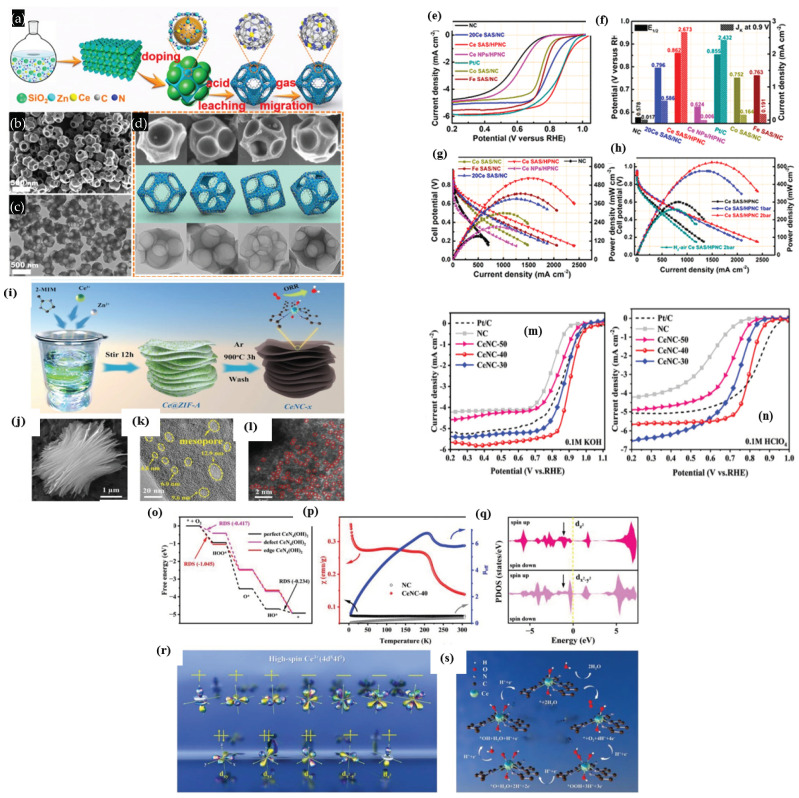
(**a**) Schematic synthesis of Ce SAC/HPNC (**b**–**d**) SEM and TEM images of Ce SAC/HPNC catalysts. (**e**) LSV curves of various Ce SAC/HPNCs. (**f**) Half-wave potential and J_k_ of various Ce SAC/HPNC catalysts. (**g**,**h**) Fuel cell characterization of the various Ce SAC/HPNC catalysts (Reproduced with permissions from Ref. [162]). (**i**) Typical representation of the synthesis of CeNC-40 catalyst. (**j**–**l**) TEM images of the CeNC-40 catalyst. (**m**,**n**) LSV curves of the CeNcs-40 catalysts in 0.1 M KOH and 0.1 M HClO4. (**o**) Free energy of the ORR on CeNCs-40. (**p**) Magnetic susceptibility and (**q**) effective magnetic moment of CeNC-40 catalyst. (**r**) Schematic representation of the electronic configuration of the high spin-state cerium atom. (**s**) Illustration of the catalytic mechanism of the ORR (Reproduced with permissions from Ref. [100]).

Though the Ce has excellent properties, such as its ability to interchange the oxidation states between Ce^3+^/Ce^4+^, it is challenging to obtain Ce in an atomically dispersed state due to its wide range of possible oxidation states from 0 to +4; therefore, Ce has the tendency to form several phases such as oxides, carbides, and metallic Ce during the high-temperature pyrolysis. Furthermore, controlling the porous nature of the catalyst is paramount to host a high density of atomically dispersed Ce atoms, which helps in exposing the high volume of the ORR active sites. Zhu et al. [162] presented highly dispersed Ce atom catalysts with a high coordination number for excellent ORR. The highly electrocatalytically active Ce SAS/HPNC catalyst was derived by a combined MOF and hard-template synthesis strategy by using SiO_2_ as a structural assisting agent, followed by acid leaching and a gas-migration process (Figure 13a). This study involves pyrolysis of the Ce-ZIF-8@SiO_2_ precursor at a relatively high temperature of 1150 °C, slightly higher than in serval studies described in the previous sections. Authors describe that, during a 1150 °C in flowing of N_2_, the surface cerium atoms of CeO_2_ were initially volatilized to generate a potential volatile cerium species which could be conveyed and captured by the defective nitrogen-rich porous carbon, resulting in an atomically dispersed Ce-SAS/HPNC catalyst. The Ce-SAS/HPNC catalyst showed excellent morphology with a highly porous rhombododecahedron single-particle (Figure 13b–d). Authors systematically established a relation between the Ce and Zn precursors and the obtained catalyst morphology. It was found that Ce’s precursor (with respect to Zn precursor) with 10–20 wt.% resulted in the traditional rhombododecahedron morphology of ZIF-8 in the Ce SAS/HPNC catalyst, whereas, with ZIF-8 and SiO_2_, a Ce precursor as high as 50 wt% has been utilized. With the SiO_2_ template strategy, the Ce-SAS/HPNC catalyst resulted in a surface area of 1095 m^2^ g^−1^ from an unusually low surface area of 61 m^2^ g^−1^ for 20Ce SAS/NC. This indicates that the adopted SiO_2_ hard-template strategy could enhance the surface area 10 times more and help host the high wt% of the Ce precursor and hence encourage the high density of Ce-N_4_-C catalysts for enhanced ORR activity. The FT-EXAFS analysis of the Ce-SAS/HPNC catalyst revealed the presence of Ce-N/O bonding at the 2.09 Å, which falls in between the bonding peaks of Ce-Cl (2.05 Å) and Ce-Cl (2.05 Å); therefore, there is a high probability that Ce might exist in the form of Ce-N/O. The ORR analysis of the Ce-SAS/HPNC catalyst in 0.1 M HClO_4_ delivered a high ORR onset potential of 1.04 V and a half-wave potential of 0.862 V vs. RHE, which are almost similar to commercial the Pt/C catalyst of 20 wt% of Pt. In addition, the Ce-SAS/HPNC catalyst also delivered an admirable performance in realistic working fuel cell conditions. The Ce-SAS/HPNC catalyst delivered a peak power density of 475 and 525 mW cm^−2^ in H_2_-O_2_ with 1 and 2 bar pressure. At 2 bar pressure, the Ce-SAS/HPNC catalyst showed a current density of 0.473 A cm^−2^ at 0.6 V (Figure 13e–h).

Though the atomically dispersed Ce atoms exhibited excellent ORR activity, there is still room to further enhance the ORR activity. The shielding effect of the inner electron shell of cerium results in the localization of the outermost 4f orbital electrons. Reshaping the coordination and geometrical structure of the active sites could lead to the transition of electrons from 4d^10^4f^1^ to 4d^8^4f^3^, leading to the formation of high-spin Ce active sites that enhance the unpaired f electrons to occupy the anti-π orbitals of O_2_ and generate optimal binding strength with ORR intermediates. To understand the importance of spin effects on ORR catalysts, it is important to understand the simple ORR process. The ORR process begins with gaseous O_2_ adsorbance on the catalyst surface and for the adsorbance of the electrons from the catalytic active site, say, a metallic site, to anti-bonding orbitals of O_2_ to occur. The high electron density of O_2_ weakens the O=O bond, allowing the O=O bond to break [163]. This entire reaction proceeds in several steps, with multiple reaction intermediates such as O*, OH*, and OOH*. During these steps, O_2_ changes its paramagnetic nature to a diamagnetic nature [164]. For efficient ORR, the catalyst surface with high spin is generally considered more favorable for O_2_ adsorption, as it helps to facilitate the spin-state matching of the adsorbed O_2_. This enhances the rapid electron transfer and ORR kinetics [165]. Therefore, the spin-state modulation of the catalyst surface could be an effective strategy to tune the ORR activity of the catalysts. In this regard, Ce spin state modulation is possible due to its nearly empty orbitals being able to accommodate high-spin electrons [166]. Based on this assumption, Zhao et al. [100] proposed a carboxylate mediated synthesis approach to tune the CeNCs catalyst toward ORR. The synthesized CeNC-40 showed an accordion-like stacked multilayer shape, with large pores between the layers to facilitate exposure of more active sites and mass transport along with an atomically dispersed, high density of Ce atoms inside the N-doped carbon matrix (Figure 13j–l). The ICP-OES analysis shows that a Ce atomic wt% as high as 7.33 is achieved without any agglomerations along with a surface area of CeNC-40 catalyst with 607 m^2^ g^−1^ with micro and mesopores in the catalysts.

The XPS analysis showed Ce-N_x_ moieties, suggesting an electronic interaction between Ce and N_x_/OH ligands, which helps in re-distributing the electronic density around the Ce atoms and hence effects the local electron and spin configuration of the Ce in the catalyst matrix. As mentioned earlier, the ratio of Ce^3+^/Ce^4+^ is an important deciding factor of ORR. This is due to the Ce^4+^ (5s^2^5p^6^)’s fully occupied electrons that are shielded with inner orbitals, whereas Ce^3+^ (5s^2^5p^6^4f^1^) has localized f-electrons which interact easily with the adsorbed O_2_ p electrons, thus promoting the effective absorption of ORR intermediates. Therefore, the atomic ratio of Ce^3+^/Ce^4+^ is an important deciding factor for Ce-based ORR catalysts. The Ce L3-edge X-ray absorption near edge structure (XANES) verified that the CeNC-40 catalyst contains Ce, predominantly in the form of Ce^3+^, which is the most active site of the catalyst. When applied as an ORR catalyst, CeNC-40 showed excellent halfwave potentials of 0.90, 0.80, and 0.7 V in alkaline, acidic, and neutral electrolytes, respectively (Figure 13m,n). The ORR activity of the CeNC-40 catalyst is gauged through theorical simulations based on the co-ordination environment derived from the EXAFS fitting result, suggesting that the coordination number of Ce-N/O is six. The theoretical simulations suggest that O_2_ adsorption occurs on the Ce-N/O active site by an end-on mode with the O-O bond length increased by 0.04Å, with a final bond length of 2.92Å on the Ce-O active site. This effective adsorption leads to d-p-f orbital hybridization and coupling, resulting in formation of *OH as RDS with an energy barrier of 0.2eV. The high-spin states of Ce are estimated by measuring magnetic susceptibility and magnetic moment of the CeNC-40 catalyst which are 5.7 and 5, respectively, which are significantly higher than those of the NC, which are 0.4 and 0, which confirms the high-spin sate of the Ce in the catalyst. PDOS analysis shows the electron insertion from the d_z_^2^ → d_x_^2^ − _y_^2^ of Ce into 4f orbitals of Ce, which causes the high-spin sate of Ce (Figure 13o–s). This electron transfer shifts the Ce electronic configuration to 4d^10^4f^1^ → 4d^8^4f^3^. The increased magnetic moment 1.95 of the Ce-NC-40 suggests that the adsorption and activation of O_2_ is elevated, with change in the spin-state of Ce promoting a direct four-electron reduction of O_2_.

**Figure 14:**
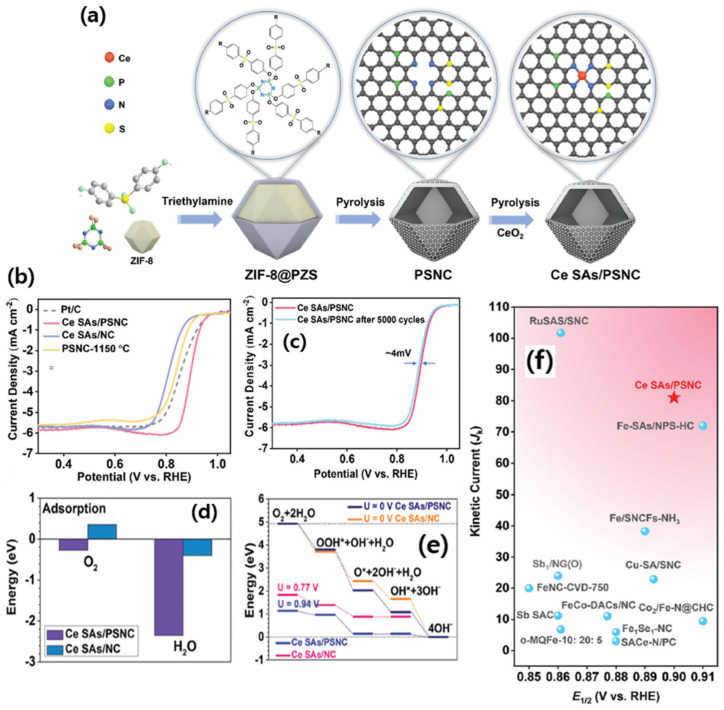
(**a**) Schematic of the synthesis procedure of Ce SAs/PSNC ORR activity of different catalysts. (**b**) Polarization curves in O_2_-saturated 0.1 m KOH solution. (**c**) Comparison of the *J*_k_ and *E*_1/2_ values between Ce SAs/PSNC and the catalysts reported recently. (**d**) LSV of Ce SAs/PSNC before and after 5000 cycles. (**e**) Adsorption energy of O_2_ and H_2_O. (**f**) The reaction energy trends of ORR formation for Ce SAs/PSNC and Ce SAs/NC (Reproduced with permission from Ref. [157]).

Ce SAs with optimized local coordination microenvironments via heteroatoms are anticipated to significantly enhance ORR performance. The introduction of N atoms into the carbon matrix, together with Ce-N_x_ active sites, are found to enhance the ORR activity. Introducing multiple heteroatoms having electron donation/withdrawing ability could create multiple electron delocalization pathways to synergistically enhance ORR activity. In a study by Du et al. [157], introducing single-atom Ce catalysts doped with C, N, and P in the carbon matrix is found to enhance the ORR activity, surpassing the effect of the Pt/C catalyst. The experimental validation and DFT insights clearly show enhanced ORR activity for Ce SAs/PSNC compared to the Ce SAs/NC catalyst (Figure 14a–f). DFT analysis of the Ce SAs/PSNC and Ce SAs/NC catalysts clearly distinguishes the contribution of P and S on the electronic distribution near Fermi energy (E_f_). It was found that the binding orbitals and electronic distribution on the Ce SAs/PSNC catalyst are larger than on the Ce SAs/PSNC catalyst due to additional contributions from the p orbitals’ S and P atoms, therefore enhancing electron delocalization and the electron transfer efficiency in Ce SAs/PSNC compared to the Ce SAs/NC catalyst. With P and S atoms in equal ratio, it was found that Ce 4f orbital electron density was closest to the E_f_, whereas for the Ce SAs/NC catalyst there is a definite gap. From DFT analysis, it was found that an applied potential of 0.94 V is required for Ce SAs/PSNC to initiate the ORR, whereas it is 0.77 V for the Ce SAs/NC catalyst, suggesting that tuning the carbon support with different heteroatoms of electron donation or electron withdrawing properties can alter the electronic charge polarization and local change and spin densities favoring the ORR. The assumptions made from DFT studies have been clearly established from the experimental point of view. The Ce SAs/PSNC catalyst is synthesized from the poly(cyclotriphospazeneco-4,4-sulfonyldiphenol) (PZS) polymerization on the ZIF-8, onto which a gas-migration strategy was applied to vaporize atomic Ce from CeO_2_ at the temperature of 1150 °C to be incorporated into NSP-doped carbon. In the ORR electrocatalytic performance, the Ce SAs/PSNC catalyst exhibited a half-wave potential of 0.90 V vs. RHE, better than the Ce SAs/NC, PSNC-C, and Pt/C catalysts, along with the highest obtained kinetic current, surpassing several non-precious metal-based electrocatalysts for ORR and enhanced stability with a loss of just 4 mV after 5000 potential cycles (Figure 14a–f).

In a unique study, Liu et al. [167] demonstrated the role of the porous structure of the catalyst and its interaction with Ce-N_4_ active sites which certainly affect the active site accessibility and enhances the interaction with O_2._ Atomically dispersed Ce SACs were synthesized via pore-confinement-pyrolysis of metal Ce/phenanthroline complexes. The detailed experimental and theoretical analysis of the Ce SA/MC catalyst revealed that pore-coupling with CeN_4_ active sites can accelerate the electron transfer, resulting in lower free energy for the RDS step during ORR. The theoretical simulations suggest clear charge redistribution in N-C and CeN_4_ active sites. In the case of the Ce SA/MC catalyst, an apparent electron transfer from CeN_4_ to mesoporous carbon was noted. The Bader charge analysis of CeN_4_ and Ce SA/MC catalysts reveals that 1.22–1.26 e^−^ were transferred from Ce/C active sites to four coordinating N atoms of CeN_4_, whereas 1.22–1.39 e^−^ have been transferred to four coordinating N atoms of CeN_4_ in a mesoporous Ce SA/MC catalyst, suggesting that the porous nature of Ce SA/MC catalyst helps in enhanced charge transfer to the active sites. The potential free energy diagram for the RDS shows that an energy barrier of 0.24 eV was noticed for the Ce SA/MC catalyst, whereas it was 0.36 eV for simple CeN_4_. These results indicate that the mesoporous nature of the catalyst certainly helps with efficient charge transfer and ORR kinetics. Similar assumptions were experimentally derived for Ce SA/MC catalysts. The Ce SA/MC catalysts exhibited a half-wave potential of 0.845 V vs. RHE, similar to the commercial Pt/C catalyst, with an electron transfer number of 3.88 and loss of 9 mV half-wave potential in a stability test by potential cycling for 5000 cycles.

The DFT analysis by Jang et al. [44] revealed the effect of the type of oxygen vacancies in the CeO_2_ (111) on the ^•^OH and ^•^OOH radical scavenging efficacy. In terms of CeO_2_ radical scavenging capacity, the generation of Ce^3+^ is synchronized with the desorption (i.e., vacancy formation) and adsorption of oxygen on the ceria surface. The vacancies created are either in triangular or linear patterns. Jang et al. [R6] investigated the effect of the CeO_2_ vacancies on the radical scavenging mechanism. The DFT studies modeled with ^•^OH and ^•^OOH show that the negatively polarized O atom of ^•^OH and ^•^OOH interacts with the high-spin state Ce and this interaction involves the spin change (decrease) on the Ce atoms. Radical oxygen would be more drawn to the cerium atoms surrounding the triangular defect, increasing the magnitude of the binding energy for stronger adsorption since it has a high-spin state and prefers to associate with other high-spin state atoms. For instance, the ^•^OOH adsorption results in a spin decrease from 1.10 to 0.86 and for ^•^OH it is 1.08 to 0.82 on the triangular defect site, lower than the spin decrease in the linear defect sites. The ^•^OH and ^•^OOH binding energies are found to be −4.54 and −3.20 eV for triangular defects, better than the values on the linear defects. The PDOS analysis reveals that the 4f band of Ce on the defective surface would play a crucial role in forming a bond with the radical species. Multiple dense peaks for the triangular defect arise from the 4f peak above the Fermi level, moving down to the below the Fermi level (−0.5 to −0 eV). More enhanced ^•^OOH radical scavenging ability is attributed to these 4f peaks around the −0.5−0 eV region in the triangular defect rather than in the linear defect. Consequently, our PDOS calculations imply that the oxygen vacancy generates the electronic spin localization on Ce atoms at the defect site, therefore generating a downshift of the Ce 4f band to below the Fermi level. The extreme scavenging capacity of ceria depends on such a change in the Ce 4f band. When developing radical scavengers against ^•^OH and ^•^OOH to improve the durability of polymer electrolyte membranes in fuel cells, it is advised to use ceria with mostly triangular defects, considering their stability and radical scavenging capacity.

In short conclusion, the pristine CeO_2_ could alone possess admirable ORR activity. In addition, the atomically dispersed Ce atoms in the form of Ce-N_4_-C active sites have shown excellent electrocatalytic activity. In this regard, using MET-6, a zinc triazolate metal–organic framework derivative, is a praising strategy which could introduce Ce atoms with atomic levels as high as 15.9% atomic percentage, successfully avoiding the generation of Ce nanoparticles. Most single-atom Ce-based catalyst studies conclude a similar point with regard to the Ce^3+^/Ce^4+^ atomic ratio, which suggests that Ce^3+^ is good at scavenging ^●^OH radicals and that Ce^4+^ is good at oxidizing H_2_O_2_. The ratio of Ce^3+^/Ce^4+^ is an important deciding factor of ORR. This is due to the Ce^4+^ (5s^2^5p^6^)’s fully occupied electrons that are shielded with inner orbitals, whereas Ce^3+^ (5s^2^5p^6^4f^1^) has localized f-electrons which interact easily with the adsorbed O_2_ p electrons, thus promoting the effective absorption of ORR intermediates. Therefore, the atomic ratio of Ce^3+^/Ce^4+^ is an important deciding factor for Ce-based ORR catalysts. In addition, it was found that the increased magnetic moment of the Ce-NC-catalysts suggests that the adsorption and activation of O_2_ is elevated with a change in the spin-state of Ce, promoting a direct four-electron reduction of O_2_. Therefore, in the Pt-, Co-, and Fe-based catalysts, the CeO_2_ could itself be an ORR active site and, in close proximity to Pt, CoN_4_, and FeN_4_ active sites, it can act as a co-catalyst in modifying the electronic nature of the major active site due to its excellent oxygen storage capacity, enhancing stability and scavenging radical species. Table 1 shows the ORR half-cell and fuel cell/Zn–air battery performance data of Ce-based catalysts [168,169,170,171,172] derived from metal–organic framework precursors.

## 7. Conclusions

Rare earth metal-based catalysts with *4f* electronic shells are popular in electrocatalysis. With its rich oxygen vacancies and affinity and facile exchange of Ce^3+^ and Ce^4+^ oxidation states, Ce is especially attractive for electrochemical reactions like oxygen reduction reaction (ORR). CeO_2_ also interacts strongly with noble metals like Pt and non-precious metals, enhancing metal/metal oxide interactions and promoting ORR by optimizing reaction intermediate adsorption. Most importantly, Ce is one of the best radical scavenging agents for fuel cell catalysts because it can interexchange Ce^3+^/Ce^4+^ oxidation states. CeO_2_ is stable in acidic conditions and improves carbon corrosion resistance in acidic electrolytes. Additionally, ceria promotes noble metal dispersion and improves O_2_ adsorption and ORR kinetics. Ceria-modified carbon compounds are effective ORR direct four-electron reduction catalysts. After a detailed analysis of the literature, it is found that Ce-based catalysts show excellent ORR activity. In the case of Pt/CeO_2_, the presence of Ce^3+^ was found to enhance the metallic ability of Pt nanoparticles and effectively inhibit the oxidation of Pt nanoparticles. The mass and specific activities of the Pt/CeO_x_/C catalysts are shown to be 10 times greater than the commercial Pt/C catalyst. The triple-phase interface structures formed from the Pt, CeO_x_, and C, are known to be responsible for the strong metal–support interactions (SMSI), contributing to the enhanced stability of Pt nanoparticles on the CeO_x_/C catalyst support. The Pt/CeO_2_-NC catalyst delivered a power density of 1.08 W cm^−2^, higher than Pt/NC and Pt/C catalysts. The enhanced ORR activity, stability, and fuel cell performance of the Pt/CeO_2_-NC catalyst was attributed to several causes, such as an effective three-phase interface structure.

In addition to the role of Ce as a co-catalyst and O_2_ buffering agent, Ce plays an important role in spatially confining and stabilizing Fe atoms. Ce doping allows charges to migrate between Fe and N elements, affecting the charge imbalance in the catalyst. This charge transfer effect lowers the d-band center value, which in turn reduces the adsorption energy of oxygen-containing intermediates in electrochemical reactions and increases the ORR electrocatalytic efficiency. The Ce 4f^1^ electrons have a high affinity for interacting with the transition metal atoms such as Fe and help in electron transfer from Ce → Fe, further enhancing the interaction of O_2_ on the Fe active sites, due to 4f^1^ localized electron transfer to the d-orbitals of the Fe active site due to their difference in electronegativity values, therefore enhancing the ORR activity of the Fe-N-C catalysts, as seen from the literature discussed inside the manuscript. The synergistic effect of ceria not only prevails in Fe-based ORR catalysts, but also in Co-based catalysts for ORR. The buffering (store/release mechanism of oxygen) capacity of CeO_2_ can be further tuned by choosing alternative Ce precursors to the traditional Ce nitrates. It was found that with a Ce_2_(OH)_4_SO_4_·2H_2_O precursor, the catalyst could generate more oxygen vacancies than by using traditional Ce precursors such as Ce nitrates. CeO_2_ also acts as a coupling metal oxide in the heterostructure catalysts with other transition metal oxides, exhibiting synergistic effects such as the heterostructure of the CO_3_O_4_-MnO_2_/C catalyst, delivering enhanced ORR activity, owing to a direct four-electron reduction of O_2_ as a result of covalent coupling between CO_3_O_4_-MnO_2_. Because of the strong electronic coupling effect, it is believed that hybridizing *p*-type Co_3_O_4_ and *n*-type CeO_2_ will increase the activity of oxygen catalysis. By combining n- and p-type materials, engineers can design and fine-tune an electrical structure that is advantageous for optimizing surface properties for enhanced ORR. Ce doping is found to be beneficial, not only for increasing the ORR activity but also for enhancing the stability of the transition metal catalysts such as the Co@NC catalyst. The addition of Ce was found to have a stability effect on the irreversible aggregation of atomic cobalt sites into Co nanoparticles, with the help of assessing the binding energy (Δ*E*_b_) between Co/Ce single atoms and carbon-based defect vacancies. Ce doping into the catalysts increases the distance between the Co atoms and hence acts as the dispersing agent and the stabilizing agent, effectively mimicking the Co aggregation and hence increasing the overall stability of the electrocatalyst. Atomically dispersed Ce atoms in the N-doped carbon showed excellent ORR activity. This is because, due to the abundant oxygen vacancies and quick transition between Ce^3+^ and Ce^4+^, the Ce itself can act as ORR active site. Ce SACs were found to have high-spin state configurations that are potentially active sites for ORR. The Ce-N-C catalysts have been shown to possess very high surface areas, as high as 900 m^2^/g. The Ce^3+^ ↔ Ce^4+^ redox couples’ ability to scavenge the peroxide/hydroperoxide radicals is confirmed from the chronoamperometric conditions and oxidation current analysis profiles of Pt/C and Ce-N-C catalysts, which show a strong scavenging ability of Ce-based SACs. These results indicate that the rare earth Ce-based catalysts are particularly promising for ORR, either in an atomically dissevered state or in combination with transition metal atoms, or with Pt that results in electrocatalytically active and stable catalysts for ORR in fuel cells and metal–air batteries.

## 8. Future Perspectives

1. Pt/CeOx/C catalysts were found to enhance the stability of the catalyst. However, there are only a few studies on the fuel cell performance analysis of the Pt/CeOx/C catalysts. At this moment, most of the studies conclude either with RDE studies and/or with single-cell performance. These catalysts could be the best alternative to the traditional Pt/c catalyst, aiming to achieve best durability. However, care should be taken while optimizing the CeO_2_ content due to its poor electronic conductivity property.

2. Fe/Co-Ce-based catalysts have the potential to be alternative catalysts for ORR. However, most of the catalysts’ ORR activity is analyzed in a traditional three-electrode system, whose output might not always be translated to realistic fuel cells and Zn–air battery applications. Therefore, it is highly essential to gauge the activity of the catalysts in terms of deliverable power density in single cell/fuel cell stacks in order to really appreciate their performance. While several studies have performed Zn–air battery performance studies, fuel cell performance studies are rare and insufficient to include their use in realistic applications, including catalyst loading optimizations, catalyst thickness optimization, and catalyst ink optimizations.

3. There is enough evidence on the effect of Ce3+/Ce4+ redox couple on the radical scavenging in fuel cells, especially CeO_2_ as additive to the nafion membrane. However, the Fe/Co-Ce-based catalyst’s radical scavenging ability in the catalyst layer is just being studied. The authors believe it is essential to investigate the ability of Fe/Co-Ce-based catalysts and their radical scavenging capacity in realistic fuel cell/Zn–air battery conditions by employing in situ studies to understand the stability of the catalysts.

4. At present, there is a lot of ambiguity in comparing Fe/Co-Ce and Ce SACs with traditional Pt/C activity, because most of the studies express the superiority of the Fe/Co-Ce and Ce SACs in terms of half-wave potentials. In order to truly compare the superiority, it is essential to express the activity of the catalyst, as recommended by the Department of Energy (DoE) regulations, such as kinetic current, mass activity of the catalysts, and the stability tests, as recommended by the DoE.

5. Few studies have investigated the role of ceria in the preparation process, which not only spatially confines Fe atoms, but also traps and stabilizes Fe atoms, resulting in high atomically dispersed Fe contents in the catalysts. However, there is no clear understanding or discussion of the confining mechanism and the kinetic control of stabilizing the Fe atoms. Also, it is essential to validate the confining mechanism by theoretical simulations.

6. From the extensive experimental output, it is concluded that the catalyst having a high content of Ce^3+^ is an important aspect for ORR. However, the precise control and abundance of a specific oxidation state in the catalyst is not well understood in the studies. Therefore, efforts need to be undertaken through novel catalyst synthesis strategies and processing strategies

7. Through DFT analysis, it is derived that CeO_2_ with a triangular defective structure is the most energetically favored, considering for structure, stability, and radical scavenging capacity. However, there is no experimental evidence regarding CeO_2_ with a triangular defective structure. Synthesizing CeO_2_ with triangular and linear defects and experimentally validating them still needs to be explored.

8. Single-atom Ce-based catalysts are only just being explored as excellent ORR catalysts, intrinsically or in combination with transition metal atoms. In particular, the high-spin states of Ce spin mechanisms are very interesting. However, at this moment, DFT studies provide very limited understanding. It is essential to investigate more on the effect of electronic spin states and the effect of magnetic moments on the ORR in detail.

9. Fe/Co-ZIF synthesis in the presence of Ce always alters the kinetic and growth of ZIF, which has been observed experimentally, where one can see the slow appearance of the milky product/precipitate of ZIFs. From this point of view, kinetic studies are required to understand the growth kinetics of Ce-ZIFs.

## Figures and Tables

**Table 1:** ORR half-cell and fuel cell data of Ce-based catalysts derived from metal–organic framework precursors.

Catalyst	ORR Onset Potential (V vs. RHE) ^a^ HClO_4_ ^b^ KOH	ORR Half-Wave Potential (V vs. RHE) ^a^ HClO_4_ ^b^ KOH	^a^ Number of Electrons (n) (from K–L Plots) ^b^ Tafel Slope (mV/dec)	Number (RRDE) of Electrons (n) and % of ^a^ H_2_O_2_ ^b^ H_2_O_-_	Potential Loss E_1/2_ (mV) Number Cycles ^b^ Loss in E_1/2_	Power Density ^a^ PEM ^b^ AEM ^c^ Zn–Air Battery mW/cm^2^	Ref.
CeGS	^b^ 0.92	^b^ 0.81	^a^ 4 ^b^ 111	n = 3.9 ^b^ 5	NR	NR	29
Ce-N-C-3	NR	^a^ 0.68	^a^ 3.91 ^b^ 97	NR	^a^ 5000 ^b^ 70	NR	31
Pt/CeO_2_-NC	NR	^a^ 0.922	NR	^a^ n = 3.94 ^a^ 0.5%	^a^ 10,000 ^b^ 10	1.08 W cm^−2^	62
0.5La-CeNC-Fe	NR	^b^ 0.870	^a^ 3.94 ^b^ 86.8	^a^ n = 3.90−3.96 ^b^ 2.26−5.55%	^a^ 1000 ^b^ 7	NR	83
Ce/Fe-NC/Fe_3_C-P	^a^ 0.94 ^b^ 0.98	^b^ 0.87 ^b^ 0.78	^b^ 167	n = 3.96–3.99 ^a^ 6%, ^b^ 3%	^a^ 10,000 ^b^ 14	^a^ 347 ^c^ 184	85
FeCeNC	NR	^b^ 0.855	^a^ 3.7 ^b^ 55	^a^ NR	^a^ 1000 ^b^ 2	^c^ 169.2	105
Fe_1_Ce_1_@N–C	^b^ 0.981	^b^ 0.867	^a^ 4 ^b^ 60	^a^ n = 3.87 ^b^ 7.1	^a^ 1000 ^b^ 40	NR	106
CeNC	^a^ 1.05 ^b^ 1.05	^a^ 0.90 ^b^ 0.80	^a^ ~4 ^b^ 80	n = 4.0 ^a^ 0.93– 1.67% ^b^ 0.41–0.70%	^a^ 10,000 ^b^ 8	^b^ 165	100
FeCe-SAD/HPNC	^a^ 0.91	^a^ 0.81	^b^ 69	^a^ n = 4 ^b^ <1.2	NR	^a^ H_2_/O_2_: 771 H_2_/air: 498	101
Ce/Fe-NCNW	NR	^b^ 0.915	^a^ 68	^b^ <2.5	^a^ 5000 ^b^ 19	^b^ 496	103
Ce-HPCN	^b^ 0.923	^b^ 0.831	^b^ 91	^b^ 5.6%	^a^ 1000 ^b^ no loss	NR	108
Co_3_O_4_@Z67-N700@CeO_2_	NR	^b^ 0.86	n = 4 ^b^ 66.8	n = 3.82–3.92 ^b^ 4.10 to 8.55%	^a^ 5000 ^b^ 6	NR	112
Ce@Co_3_O_4_/CNFs	NR	^b^ 0.81	n = 4 ^b^ 85.8	NR	NR	^c^ 97.7	116
Co_3_O_4_/CeO_2_@Co/*N*-CNF	^b^ 0.88	^b^ 0.75	^a^ 127.6	NR	NR	NR	121
CeCo-NxCC	^b^ 0.95	^b^ 0.85	n = 3.7	n = 3.98 ^b^ 9	^a^ 8000 ^b^ 10.1	^c^ 114	130
Co_9_S_8_/CeO_2_/Co-NC	NR	^b^ 0.875	n = 4.0	n = 3.80 ^b^ 8.3	^a^ 2000 ^b^ 10	^c^ 164	138
CeO_2_@N-C-900	^b^ 1.003	^b^ 0.908	^a^ n = 3.97	NR	^a^ 3000 ^b^ no loss	NR	142
CeO_2_–CN–800	^b^ 0.90	^b^ 0.84	^a^ n = ~4	n = ~4 ^b^ 10	NR	^c^ 65	143
Ce-N_4_O_2_	NR	^b^ 0.87	^a^ n = ~4 ^b^ 76	NR	^a^ 30,000 ^b^ 11	^b^ 180	159
Ce-N-C	NR	^b^ 0.89	^a^ n = 3.94 ^b^ 62	n = ~4 ^b^ 4	^a^ 10,000 ^b^ 190	NR	160
Ce SAS/HPNC	^a^ 1.04	^a^ 0.862	^a^ 66	NR	NR	H_2_/O_2_ 525 H^2^/air: 250 W	162
CeO_2_/Co@N–C	^b^ 0.998	^b^ 0.934	^a^ n = 4	NR	NR	^c^ 103	168
CeO_2_@CoSe_2_-NCs	NR	^b^ 0.76	^a^ n = 4 ^b^ 86	n = 3.8–3.9 ^b^ 9	NR	^c^ 153	169
4.8% Ce-MnO_2_/C	^b^ 0.872	^b^ 0.783	n = 3.97 ^b^ 90	n = 4 ^b^ 1.0–2.7	NR	348 (Al–air)	170
CeO_2_/rGO	^b^ 0.91	NR	^a^ n = 3.3–3.5	NR	NR	NR	171
CeCNC_F_	^b^ 0.86	^b^ 0.70	n = 3.99	^b^ 0.06	^a^ 3000 ^b^ 25	NR	172

NR = Not reported.
